# The developmental and iron nutritional pattern of PIC1 and NiCo does not support their interdependent and exclusive collaboration in chloroplast iron transport in *Brassica napus*

**DOI:** 10.1007/s00425-020-03388-0

**Published:** 2020-04-15

**Authors:** Hong Diep Pham, Sára Pólya, Brigitta Müller, Kálmán Szenthe, Máté Sági-Kazár, Barbara Bánkúti, Ferenc Bánáti, Éva Sárvári, Ferenc Fodor, László Tamás, Katrin Philippar, Ádám Solti

**Affiliations:** 1 Department of Plant Physiology and Molecular Plant Biology, Institute of Biology, ELTE Eötvös Loránd University, Budapest, Hungary; 2 RT-Europe Nonprofit Research Ltd., Mosonmagyaróvár, Hungary; 3 Center for Human - and Molecular Biology, Plant Biology, Saarland University, Saarbrücken, Germany

**Keywords:** Chloroplast, Iron deficiency, Iron homeostasis, Leaf development, Supraoptimal iron nutrition

## Abstract

**Main conclusion:**

The accumulation of NiCo following the termination of the accumulation of iron in chloroplast suggests that NiCo is not solely involved in iron uptake processes of chloroplasts.

**Abstract:**

Chloroplast iron (Fe) uptake is thought to be operated by a complex containing permease in chloroplast 1 (PIC1) and nickel–cobalt transporter (NiCo) proteins, whereas the role of other Fe homeostasis-related transporters such as multiple antibiotic resistance protein 1 (MAR1) is less characterized. Although pieces of information exist on the regulation of chloroplast Fe uptake, including the effect of plant Fe homeostasis, the whole system has not been revealed in detail yet. Thus, we aimed to follow leaf development-scale changes in the chloroplast Fe uptake components *PIC1*, *NiCo* and *MAR1* under deficient, optimal and supraoptimal Fe nutrition using *Brassica napus* as model. Fe deficiency decreased both the photosynthetic activity and the Fe content of plastids. Supraoptimal Fe nutrition caused neither Fe accumulation in chloroplasts nor any toxic effects, thus only fully saturated the need for Fe in the leaves. In parallel with the increasing Fe supply of plants and ageing of the leaves, the expression of *BnPIC1* was tendentiously repressed. Though transcript and protein amount of *BnNiCo* tendentiously increased during leaf development, it was even markedly upregulated in ageing leaves. The relative transcript amount of *BnMAR1* increased mainly in ageing leaves facing Fe deficiency. Taken together chloroplast physiology, Fe content and transcript amount data, the exclusive participation of NiCo in the chloroplast Fe uptake is not supported. Saturation of the Fe requirement of chloroplasts seems to be linked to the delay of decomposing the photosynthetic apparatus and keeping chloroplast Fe homeostasis in a rather constant status together with a supressed Fe uptake machinery.

**Electronic supplementary material:**

The online version of this article (10.1007/s00425-020-03388-0) contains supplementary material, which is available to authorized users.

## Introduction

Iron is an essential micronutrient for all organisms. Since its availability is limited due to environmental factors for many land plants, including agricultural crops, Fe deficiency is among the most important challenges of the agriculture. Due to its versatile redox properties under biological conditions, Fe participates in various redox reactions in plants including the photosynthetic and the respiratory electron transport chains (Muneer et al. 2014), and is also involved in DNA and Chl biosynthesis (Briat and Lobréaux 1997; Rout and Sahoo 2015). In shoot tissues, 80–90% of cellular Fe is found in chloroplasts (Terry and Abadía 1986; Roschzttardtz et al. 2013), 60% of which is found in thylakoid membranes (Castagna et al. 2009). In chloroplasts, the majority of Fe incorporates into heme groups and Fe–S clusters according to Mössbauer spectroscopy measurements (Solti et al. 2012). Fe is required for the proper function of sulphate and nitrate assimilation, the elimination of reactive oxygen species by Fe-superoxide dismutase and ascorbate peroxidase and for the operation of photosynthetic electron transport. Fe deficiency decreases the Fe content of chloroplasts. Limitation in the availability of Fe leads to a significant decrease in the amount of Fe–S cluster biogenesis and sulphate assimilation pathway components, ascorbate peroxidase, cytochrome *b*_*6*_*/f* complex units PetA and PetC, photosystem I component PsaC and PsaD and soluble ferredoxin (Hantzis et al. 2018). Fe deficiency also induces significant alterations in the PSII complex organization (Basa et al. 2014). Since Fe deficiency affects the photosynthetic activity, it also reduces the crop yield (Wu et al. 2010). In contrast, slight excess of Fe does not cause significant alterations in the composition and function of the photosynthetic apparatus (Sárvári et al. 2011). Non-complexed Fe ions can be highly toxic for plants. Free ferrous ions can produce hydroxyl radicals via Fenton reactions (Halliwell and Gutteridge 1992). This surely leads to the alteration in photosynthetic efficiency and results in cellular damages (Briat et al. 2010). Therefore, Fe acquisition, translocation and storage are strictly controlled to maintain Fe homeostasis (Jeong and Guerinot 2009; Zhu et al. 2016).

In contrast to the well-characterized Fe uptake and translocation in roots (Kobayashi and Nishizawa 2012; Curie and Mari 2017), current knowledge on Fe uptake of mesophyll cells and their organelles is still scarce (Vigani et al. 2019). The cellular Fe trafficking and the Fe uptake into the organelles is undiscovered in many respects. Since Fe–S clusters are synthetized in the mitochondria and transported to the cytoplasm, mitochondria might play an important role in the signalling of the Fe status in mesophyll cells (Vigani and Hanikenne 2018). Concerning the chloroplast Fe acquisition, a number of Fe uptake-related proteins have been identified in the past decade in *Arabidopsis thaliana* (for overview, see López-Millán et al. 2016). In general, chloroplasts seem to perform a reduction-based Fe uptake strategy operated by the essential chloroplast ferric chelate reductase 7 and two membrane transport proteins, permease in chloroplast 1 (PIC1) and nickel–cobalt transporter (NiCo) in *Arabidopsis* (Duy et al. 2007, 2011; Jeong et al. 2008; López-Millán et al. 2016). Although NiCo has not been characterized at the protein level yet, PIC1 has been described as a 21-kDa protein of the Translocon at Inner Chloroplast envelope (TIC21; Teng et al. 2006). It seems to have an overlapping function as central components of the import channel with 20 kDa protein of the translocon at inner chloroplast envelope protein (Richardson and Schnell 2019). TIC21 seems to have a role in the proper assembly of the protein translocon complex in the inner membrane (Shi and Theg 2013). However, *pic1/tic21* mutants show impaired chloroplast development and iron transportation, but the protein import remains intact (Duy et al. 2007; Kovács-Bogdán et al. 2010). A multiple antibiotic resistance protein (MAR1; also known as iron regulated protein 3, IREG3) is also found in the chloroplast envelope membranes (Conte et al. 2009). Both in silico and recent experimental data suggest that MAR1/IREG3 has a dual localisation in chloroplasts and mitochondria (Schwacke et al. 2003; Zhang et al. 2018). Yellow stripe-like family Fe–nicotianamine complex transporters, Yellow Stripe-like 4 and 6, are described to play a potential role in Fe release from protoplastids of seeds (Divol et al. 2013). Recently, it was excluded that Yellow Stripe-like family transporters would have a significant role in the Fe acquisition of chloroplasts that neither utilize Fe–nicotianamine complexes in their Fe uptake nor *Yellow Stripe-like 4* showed expression in leaves in *Brassica napus* (Müller et al. 2019). In *Arabidopsis*, ABC transporters, especially the non-intrinsic ABC protein 14, also have a role in chloroplast Fe homeostasis (Shimoni-Shor et al. 2010; Voith von Voithenberg et al. 2019) and the presence of Mitoferrin-like 1 protein is predicted in chloroplasts, too. The expression of *Mitoferrin-like 1* is up-regulated by the excess of Fe (Tarantino et al. 2011).

Among chloroplast Fe acquisition-related proteins, the role of PIC1 proved to be essential. PIC1 was first described to contribute delivering Fe into the chloroplasts across the inner envelope membrane (Duy et al. 2007, 2011; Gong et al. 2015). Orthologues of *Arabidopsis thaliana PIC1* have been already identified in tomato (*Solanum lycopersicum*), maize (*Zea mays*) and sorghum (*Sorghum bicolor*). Its expression proved to be also sensitive to drought and salt stresses (Filiz and Akdudak 2019). Duy et al. (2011) reported that the overexpression of *PIC1* in *Arabidopsis* significantly increased the Fe content in chloroplasts and also resulted in leaf chlorosis as well as increase in the oxidative stress. In contrast to seeds, ferritins are known to accumulate in chloroplasts of leaves only under heavy Fe excess and sequester free Fe to prevent oxidative stress (Ravet et al. 2009; Briat et al. 2010). Nevertheless, the *PIC1*-overexpressing lines shared high level of similarities to *ferritin* knock-out mutants (Duy et al. 2011), most likely resulting from elevated levels of toxic free Fe in both mutant lines (*PIC1* overexpressing and *ferritin* knock-out, respectively). Since the reduction-based Fe uptake dominates the chloroplast Fe uptake processes, and the uptake of Fe–nicotianamine complexes is negligible in the chloroplasts of dicots (Solti et al. 2012; Müller et al. 2019), PIC1s have prime importance in the uptake of non-complexed free ferrous Fe. PIC1 interacts with NiCo, a plant member of prokaryotic nickel–cobalt transporter family (Duy et al. 2011; López-Millán et al. 2016). NiCo bears a special metal-binding domain (Eitinger et al. 2005). According to the model of Duy et al. (2011), a hypothetical metal translocon, consisting of PIC1 and NiCo units (PIC–NIC) should be responsible for chloroplast Fe uptake. Here PIC1 would act as ferrous ion (Fe^2+^) permease and NiCo might sense or bind the metal ions (López-Millán et al. 2016) and transfer Fe^2+^ subsequently to PIC1 (Vigani et al. 2019). Nevertheless, the contribution of NiCo in the Fe uptake has not been characterized yet.

MAR1/IREG3 may be an opportunistic gateway of multiple antibiotics but it may also act in the Fe homeostasis of chloroplasts by transporting nicotianamine, or Fe complexes, such as Fe–nicotianamine (Vigani et al. 2019). Aminoglycoside antibiotics use the polyamine uptake system to enter into eukaryotic cells (van Bambeke et al. 2000). Since MAR1/IREG3 likely transports aminoglycoside compounds into plastids and nicotianamine is a polyamine-like metabolite (Curie et al. 2009), MAR1/IREG3 may contribute to the nicotianamine transport of plastids and thus to the maintenance of the Fe homeostasis (Conte et al. 2009; Conte and Lloyd 2010). The level of *MAR1/IREG3* transcripts increased upon Fe starvation (Yang et al. 2010). On the other hand, *MAR1/IREG3*-overexpressing plants suffer from chlorosis that can be rescued by surplus Fe nutrition (Conte et al. 2009; Zhang et al. 2018).

Although Fe content and homeostasis in chloroplasts is essential to maintain elementary physiological functions such as the operation of the photosynthetic electron transport, regulation of Fe homeostasis and Fe transport processes in chloroplasts are hardly known under alterations of the Fe nutrition of plants. Similarly, we also lack information, how the Fe transport machinery, development and ageing of photosynthetic tissues are altered by the Fe homeostasis of chloroplasts. Thus, here we performed combined physiological and transcript amount measurements through and following the development of leaves to specify the contribution of yet uncharacterized NiCo to the Fe transport processes of plastids.

## Materials and methods

### Plant material and growth conditions

Oilseed rape (*Brassica napus* L. cv. DK Exquisite) seeds were a kind gift of Ing. agr. Rudolf Solti (Müller et al. 2019) and germinated at moderate light. Seven-day-old seedlings were transferred to and grown on half-strength Hoagland solution [2.5 mM Ca(NO_3_)_2_, 2.5 mM KNO_3_, 1.0 mM MgSO_4_, 0.5 mM KH_2_PO_4_, 0.16 μM CuSO_4_, 9.2 μM MnCl_2_, 0.38 μM ZnSO_4_, 0.24 μM Na_2_MoO_4_, 23.12 μM H_3_BO_3_ and 20 μM Fe(III)-EDTA as Fe source] in 12 l containers under controlled conditions [14/10 h light (120 µmol photons m^−2^ s^−1^ PPFD)/dark periods, 24/22 °C and 75/80% relative humidity] until reaching the four-leaf stage. To induce Fe deficiency (ΔFe), four-leaf stage plants were transferred to an Fe-free and 0.5% (w/v) CaCO_3_-containing nutrient solution. As for supraoptimal Fe nutrition (+ Fe), four-leaf stage plants were further cultivated on nutrient solution containing 100 µM Fe^(III)^–EDTA. As for optimal Fe nutrition, plants were further cultivated according to the pre-cultivation parameters. Nutrient solution was refreshed every 1 week. Leaves developed partially before and under the treatment (further referred to as 4th and 6th leaves, respectively) were monitored during the 35-day-treatment time. Growth of leaves was followed by calculating the leaf area comparing the weight of leaf discs with the weight of the whole leaves used in samplings.

### Chlorophyll *a* fluorescence induction

Fluorescence induction measurements were carried out with intact leaves using a PAM 101–102-103 chlorophyll fluorometer (Walz, Effeltrich, Germany). Leaves were dark-adapted for 30 min. The *F*_0_ level of fluorescence was determined by switching on the measuring light with modulation frequency of 1.6 kHz and PPFD less than 1 µmol m^−2^ s^−1^ after 3 s illumination by far-red light to eliminate reduced electron carriers. The maximum fluorescence yields, *F*_m_ in the dark-adapted state and *F*_m_ʹ in light-adapted state, were measured by applying a 0.7-s pulse of white light (PPFD of 3500 µmol photon m^−2^ s^−1^, light source: KL 1500 electronic, Schott, Mainz, Germany). For quenching analysis, actinic white light (PPFD of 100 µmol photon m^−2^ s^−1^, KL 1500 electronic) was provided. Simultaneously with the onset of actinic light, the modulation frequency was switched to 100 kHz. The steady-state fluorescence of light-adapted state (*F*_s_) was determined when no change was found in *F*_m_ʹ values between two white light flashes separated by 100 s. Equations of Hendrickson et al. (2005) were used for assessing the excitation energy allocation in all samples as follows:

$${\Phi }_{\mathrm{P}\mathrm{S}\mathrm{I}\mathrm{I}}=\left(1-\frac{{F}_{\mathrm{S}}}{{F}_{\mathrm{m}}{^{\prime}}}\right)\mathrm{*}\left(\frac{\frac{{F}_{\mathrm{v}}}{{F}_{\mathrm{m}}}}{\frac{{F}_{\mathrm{v}\mathrm{M}}}{{F}_{\mathrm{m}\mathrm{M}}}}\right),$$

$${\Phi }_{\mathrm{N}\mathrm{P}\mathrm{Q}}=\left(\frac{{F}_{\mathrm{s}}}{{F}_{\mathrm{m}}{^{\prime}}}-\frac{{F}_{\mathrm{s}}}{{F}_{\mathrm{m}}}\right)\mathrm{*}\left(\frac{\frac{{F}_{\mathrm{v}}}{{F}_{\mathrm{m}}}}{\frac{{F}_{\mathrm{v}\mathrm{M}}}{{F}_{\mathrm{m}\mathrm{M}}}}\right),$$

$${\Phi }_{\mathrm{f},\mathrm{D}}=\left(\frac{{F}_{\mathrm{s}}}{{F}_{\mathrm{m}}}\right)\mathrm{*}\left(\frac{\frac{{F}_{\mathrm{v}}}{{F}_{\mathrm{m}}}}{\frac{{F}_{\mathrm{v}\mathrm{M}}}{{F}_{\mathrm{m}\mathrm{M}}}}\right),$$

$${\Phi }_{\mathrm{N}\mathrm{F}}=1-\left(\frac{\frac{{F}_{\mathrm{v}}}{{F}_{\mathrm{m}}}}{\frac{{F}_{\mathrm{v}\mathrm{M}}}{{F}_{\mathrm{m}\mathrm{M}}}}\right),$$

where* Φ*_PSII_ the photochemical efficiency of functional PSII centres; *Φ*_NPQ_ ΔpH dependent, xanthophyll cycle-coupled non-photochemical quenching; *Φ*_f,D_ fluorescence/thermal dissipation of the absorbed energy; *Φ*_NF_ the thermal dissipation by inactive PSII centres. *F*_vM_/*F*_mM_ was applied as the mean of *F*_v_/*F*_m_ values of control (quasi-non-inhibited) plants grown under optimal Fe nutrition according to Solti et al. (2014).

### Isolation of chloroplasts

Chloroplast isolation was performed according to Solti et al. (2012). Leaves were homogenized in isolating buffer (50 mM HEPES–KOH, pH 7.0, 330 mM sorbitol, 2 mM EDTA, 2 mM MgCl_2_, 0.1% (w/v) BSA, 0.1% (w/v) Na-ascorbate) for 2 × 3 s in a 100-ml Waring Blender container. The homogenate was filtered through four layers of gauze and two layers of Miracloth™ (Calbiochem-Novabiochem, San Diego, CA, USA). Chloroplasts were pelleted by centrifugation with 1600*g* at 4 °C for 5 min in a swing-out rotor. The pellet was re-suspended in washing buffer (50 mM HEPES–KOH, pH 7.0, 330 mM sorbitol, 2 mM MgCl_2_), layered on the top of a stepwise sucrose gradient (50 mM HEPES–KOH, pH 7.0, 20/45/60% sucrose, 2 mM EDTA, 2 mM MgCl_2_), and centrifuged at 2000*g* for 20 min. Intact chloroplasts were collected from the 45/60% sucrose interface. After a fivefold dilution with washing buffer, the plastid fraction was centrifuged at 2500*g* for 5 min and the pellet re-suspended in washing buffer. The number of chloroplasts was determined in a Bürker chamber by light microscopy (Nikon Optiphot-2, equipped with a Nikon D*70* camera). Chloroplasts were counted using ImageJ software (rsbweb.nih.gov/ij/) with Cell Counter plugin. Intactness of chloroplasts was determined as in Solti et al. (2016) and Müller et al. (2019) comparing the Rubisco large subunit (RbcL; stroma marker) to light harvesting complex II apoprotein (apoLHCII; thylakoid membrane marker) ratio in the chloroplast suspensions to that of in the intact leaves (Suppl. Fig. S1). Both intact leaf tissue and purified chloroplast suspensions were solubilized in 62.5 mM Tris–HCl (pH 6.8), 2% SDS, 2% DTT, 10% glycerol and 0.001% bromophenol blue at room temperature for 30 min. Proteins were separated in 10–18% gradient polyacrylamide gels in a MiniProtean apparatus (Bio-Rad) using a constant current of 20 mA per gel at 6 °C. Protein concentration of samples following a colloidal Coomassie staining was determined by comparing the area densities using Phoretix 4.01 software (Phoretix International, Newcastle upon Tyne, UK). Intactness of the chloroplasts was 88.8 ± 3.1% in average.

### Determination of Chl and iron content

Chl contents were either determined from leaf disks or from isolated chloroplasts in 80% (v/v) acetone extracts by a UV–VIS spectrophotometer (Shimadzu, Kyoto, Japan) using the absorption coefficients of Porra et al. (1989).

To measure Fe content, dried leaf material was ground, digested and solubilized in 62.5 mM Tris–HCl (pH 6.8), 2% SDS and 2% DTT. After adding 1% SDS and 1% DTT to the washing buffer, chloroplasts were solubilized for 30 min at room temperature. Non-solubilized material (starch) was removed by a centrifugation at 10,000*g* for 5 min. Fe content was determined after adding 100 µM ascorbic acid and 300 µM bathophenanthroline disulphonate disodium salt (Sigma) at 535 nm by UV–VIS spectrophotometer (Shimadzu) using extinction coefficient of 22.14 mM^−1^ cm^−1^ for Fe^(II)^–bathophenanthroline disulphonate complex (Smith et al. 1952). Background was determined from blank samples measured without the addition of bathophenanthroline disulphonate.

### RNA extraction and cDNA synthesis

Approximately 100 mg of fresh leaf stored at − 80 °C was subjected to total RNA extraction using TRI reagent® (Sigma). Tissue samples were homogenized in 1 ml of TRI reagent. 0.2 ml chloroform per ml of TRI reagent was used to separate mRNA according to the manufacturer’s instruction. The nucleic acid pellet was washed in 75% ethanol, dried at RT and dissolved in 50 µl diethyl pyrocarbonate-treated water. RNA concentration was quantified by a Nanodrop ND-1000 spectrophotometer (Thermo-Fisher Scientific). Residual genomic DNA contamination was eliminated by RNase-free DNase I (Thermo-Fisher Scientific) treatment. Reverse transcription of the RNA pool was performed using random hexameric oligonucleotides by RevertAid Reverse Transcriptase (Thermo-Fisher Scientific) according to the manufacturer’s instruction. cDNA libraries were stored at − 80 °C to further applications.

### Identification of *Brassica* orthologs of the *Arabidopsis PIC1*, *NiCo* and *MAR1* genes

To identify putative homologs the genes of interest in *Brassica napus*, *AtPIC1* (*At2g15290*), *AtNiCo* (*At2g16800*) and *AtMAR1* (*At5g26820*) sequences were used as initial protein queries against Brassica Database (https://brassicadb.org/brad) to blast protein sequences. Reciprocal blasts of the identified sequences were performed against *Arabidopsis* transcripts in TAIR database (https://www.arabidopsis.org). Three single copy genes encoding *Brassica* orthologues of the *Arabidopsis* queries were identified as *BnPIC1* (*Bra03640*9; score: 461, e^−130^; 1.131 kbp sequence on A07 chromosome from 2,426,016 to 2,427,146 [minus strand]; reciprocal best hit: *At2g15290.1*; score: 343, e^−92^), *BnNiCo* (*Bra037287*; score: 494; e^−140^; 1.223 thousand base pairs [kbp] sequence on A09 chromosome from 4,511,382 to 4,512,604 [plus strand]; reciprocal best hit: *At2g16800.1*; score: 962; e^0^) and *BnMAR1* (*Bra020559*; score: 963; e^0^; 3.397 kbp sequence on A02 chromosome from 24,578,588 to 24,581,984 [minus strand]; reciprocal best hit: *At5g26820*.*1*; score: 365; 3 × e^−99^) orthologues of *At2g15290*, *At2g16800* and *At5g26820*, respectively. According to the target sequences, specific primers were designed at exon–exon border sites to avoid genomic contamination.

### Expression analysis

For accurate representation and reliable analysis of target gene expression, a robust normalization of qRT-PCR data with suitable internal control genes is paramount (Czechowski et al. 2005). *ß*-Tubulin (XM_009125342.1) and 18 s rRNA (KT225373) coding sequences were chosen to correct the non-specific variations because the differences in the amount and quality of the starting RNA/cDNA samples can affect the efficiency of qRT-PCR results (Andersen et al. 2004). Primer sequences are listed in Table 1. All target primer pair sequences amplified single PCR products of expected sizes with optimal annealing temperature and primer concentration. Efficiency measurements and qRT-PCR reactions were performed by StepOnePlus Real-Time PCR system (Applied Biosystems, Foster City, CA, USA) with the StepOne™ v.2.2.3 software. The primer specificity was confirmed by the presence of a sharp peak during melt curve stage together with 2% agarose gel electrophoresis of PCR products. A standard curve was generated using seven points of twofold serial dilutions of cDNA template to calculate the PCR efficiencies of target genes. Efficiency of each gene was estimated from the slope of a linear regression model and ranged from 1.8 to 2.05.

**Table 1:** Oligonucleotide primers used in the expression analysis of chloroplast Fe homeostasis-related transporters *PIC1*, *NiCo* and *MAR1*

Gene	Genbank accession	*Arabidopsis* ortholog	Primers	Primer sequences	Melting *T*_m_ (°C)	Efficiency	Product size (base pairs)
*BnPIC1*	*Bra03640*9	*At2g15290*	fw	5′- TGCGGTCACTACTCTTGC -3′	59	2.05	161
			rev	5′- GATGGTGGCTCTCCTCTTC -3′			
*BnNiCo*	*Bra037287*	*At2g16800*	fw	5′- CTTCCGCCACAATCCTTC -3′	59	1.88	122
			rev	5′- CATAACTCCGCACGATCC -3′			
*BnMAR1*	*Bra020559*	*At5g26820*	fw	5′-GGCTCTTCTCAGACAATCTCC-3′	59	1.90	98
			rev	5′-TGCGAACTCCAGACAAACC-3′			
18S rRNA	*KT225373*	*At2g16590*	fw	5′- GCATTCGTATTTCATAGTCAGAGGTG-3′	61	1.87	192
			rev	5′- CGGAGTCCTAAAAGCAACATCC-3′			
*ß*-tubulin	*XM_009125342.1*	*At4g20890*	fw	5′- TCSATCCAGGARATGTTCAGG-3′	59	1.91	148
			rev	5′- ACTCTGCAACAAGATCATTCATG-3′			

To identify the differential expressions among samples by qRT-PCR, 100 × diluted cDNA samples, 1.0 µM gene-specific primers and 2 × diluted SYBR Green reagent (Luminaris Color HiGreen High ROX, Thermo-Fisher Scientific) were used in the final volume of 15 µl. All cDNA samples were freshly diluted before qRT-PCRs. Samples were mixed from the leaves of identical leaf storeys of plant individuals of the same treatment (biological repetitions) in one set of experiment. The qRT-PCR program was set up as follows: a predigest step of 50 °C for 2 min, a 95 °C initial denaturation step for 10 min, 40 cycles of 95 °C for 15 s (denaturation), T_m_ for 20 s (annealing), 72 °C for 20 s (extension), and final melt curve stage. Quantification of the normalized relative transcript level of specific genes was performed according to the method of Pfaffl (2001).

### Relative quantification of proteins by immunoblot

Chloroplasts were solubilized in 1 ml of 62.5 mM Tris–HCl (pH 6.8), 2% SDS, 2% DTT, and 10% glycerol. Solubilization of chloroplast samples was also performed according to Duy et al. (2007) but replacing 100 µM phenylmethylsulfonyl fluoride with 250 μg ml^−1^ Pefabloc. The mixture was incubated at room temperature for 30 min. Non-solubilized material was removed by centrifugation at 10,000 g for 5 min, and 0.001% bromophenol blue was added. Solubilized proteins were run on 10–18% gradient acrylamide gels containing 0.1% SDS (SDS-PAGE; Laemmli 1970) and 10% glycerol in a MiniProtean apparatus (BioRad) using a constant current of 20 mA per gel slab (surface 1 cm^2^) at 6 °C. Proteins separated by SDS-PAGE were transferred to Amersham™ ProtranTM Premium 0.2 µm nitrocellulose blotting membranes (GE Healthcare) in a 25 mM Tris (pH 8.3), 192 mM glycine, 20% (v/v) methanol and 0.02% (m/v) SDS at 6 °C using constant voltage of 90 V for 3 h.

Immunoblot against BnNiCo and BnPIC1 proteins was performed with rabbit polyclonal antibodies directed against NiCo from *Pisum sativum* and against PIC1 from *Arabidopsis* (see Duy et al. 2007, 2011). Polyclonal antiserum against the recombinant, C-terminal part of the PsNiCo protein (amino acids 236–375) was raised in rabbits (Pineda Antibody Service, Berlin, Germany). To identify BnNiCo proteins, rabbit polyclonal antibodies against light-harvesting complex II apoprotein (apoLHCII; a gift from Dr. Udo Johanningmeier, Bochum Universität, Germany) were used to detect BnLHCII and to distinguish the specific bands of BnNiCo and BnPIC1. Antibodies were dissolved in 20 mM Tris–HCl (pH 7.5), 0.15 M NaCl and 1% gelatine (1:10,000 dilution). Following the horizontal transfer of the protein, membranes were blocked by 3% gelatine solved in 20 mM Tris–HCl (pH 7.5), 0.15 M NaCl. Horseradish peroxidase-conjugated goat-anti-rabbit IgG (BioRad) was used to detect bands following the manufacturer’s instructions. The amount of proteins was counted using Phoretix 4.01 software (Phoretix International). The normalization of protein quantification was based on the same amount of total chloroplast proteins.

### Statistical analysis

Corresponding measurements were repeated in three independent sets of plant material (independent experiments). Each set of plant material consisted of ten individual of identical phenology per Fe nutrition treatment per experiment. Chlorophyll and chloroplast Fe contents were measured as triplicates per treatment in three independent experiments. Fluorescence induction measurement was performed on the same treated plants during the experimental period in three biological replicates in each three independent experiments. Two parallel RNA samples (technical replicates) were isolated from each three independent experiments (biological replicates). Quantitative RT-PCR analysis of genes and samples was processed in technical triplicates to confirm the stable expression of genes of interest. Total chloroplast proteins were isolated and later processed from two technical replicates of each treatment in three independent experiments (biological replicates).

Unpaired Student’s *t* tests and one-way ANOVAs with Tukey–Kramer post hoc tests were performed on data using InStat v. 3.00 (GraphPad Software, Inc., San Diego, CA, USA). The term ‘significantly different’ mean that the similarity of samples is *P* < 0.05. Origin v. 6.01 (Origin Lab Co., Northampton, MA, USA) was used to fit mathematical functions on data points.

## Results

### Changes in physiological parameters of leaves

Development of the leaves was dependent on the Fe nutrition status of the plants (Suppl. Fig. S2). Fe deficiency inhibited the leaf area expansion markedly in both 4th and 6th leaves, but supraoptimal Fe nutrition did not cause significant alterations in the area expansion pattern of leaves compared to optimal Fe nutrition. Under both optimal and supraoptimal Fe nutrition, leaves reached their maximal area at the (14–)21th day of treatment, whereas under Fe deficiency, 4th leaves performed no area growth during the time of treatment. The area expansion of 6th leaves became complete by the 28th day of treatment in all experimental groups.

**Fig. 1:**
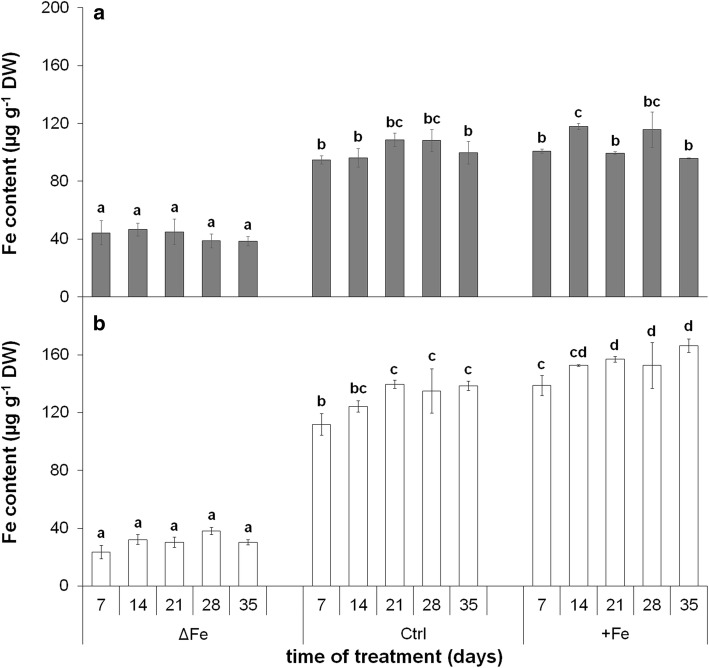
Changes in the Fe content of 4th (**a**) and 6th (**b**) leaves during the time of treatment. ∆Fe, Fe deficiency; Ctrl, optimal Fe nutrition (control); + Fe, supraoptimal Fe nutrition. Error bars represent SD values. To compare the differences, one-way ANOVAs were performed with Tukey–Kramer post hoc tests on the treatments (*P* < 0.05; *n* = 3 × 3 [biological × technical])

Leaf Fe content was strongly reduced by the limitation of Fe nutrition in both 4th and 6th leaves without any increase during the time of treatment (Fig. 1). Fe content in 4th and 6th leaves of Fe-deficient plants on the 28th day of treatment was 35.8 ± 4.4% and 28.2 ± 1.7% of the corresponding leaves in plants grown under optimal Fe nutrition. Fe content in 4th leaves of plants grown under optimal and supraoptimal Fe nutrition did not show significant differences. Under both growth conditions, the Fe content at the 35th day of the treatment was tendentiously lower than that at the 28th day. In contrast, there was a gradual increase in the Fe content of 6th leaves during the whole experimental period. The Fe content of 6th leaves with supraoptimal Fe nutrition was significantly higher during the time of treatment, 120.2 ± 3.4% of the corresponding leaf of optimal iron nutrition on the 35th day.

**Fig. 2:**
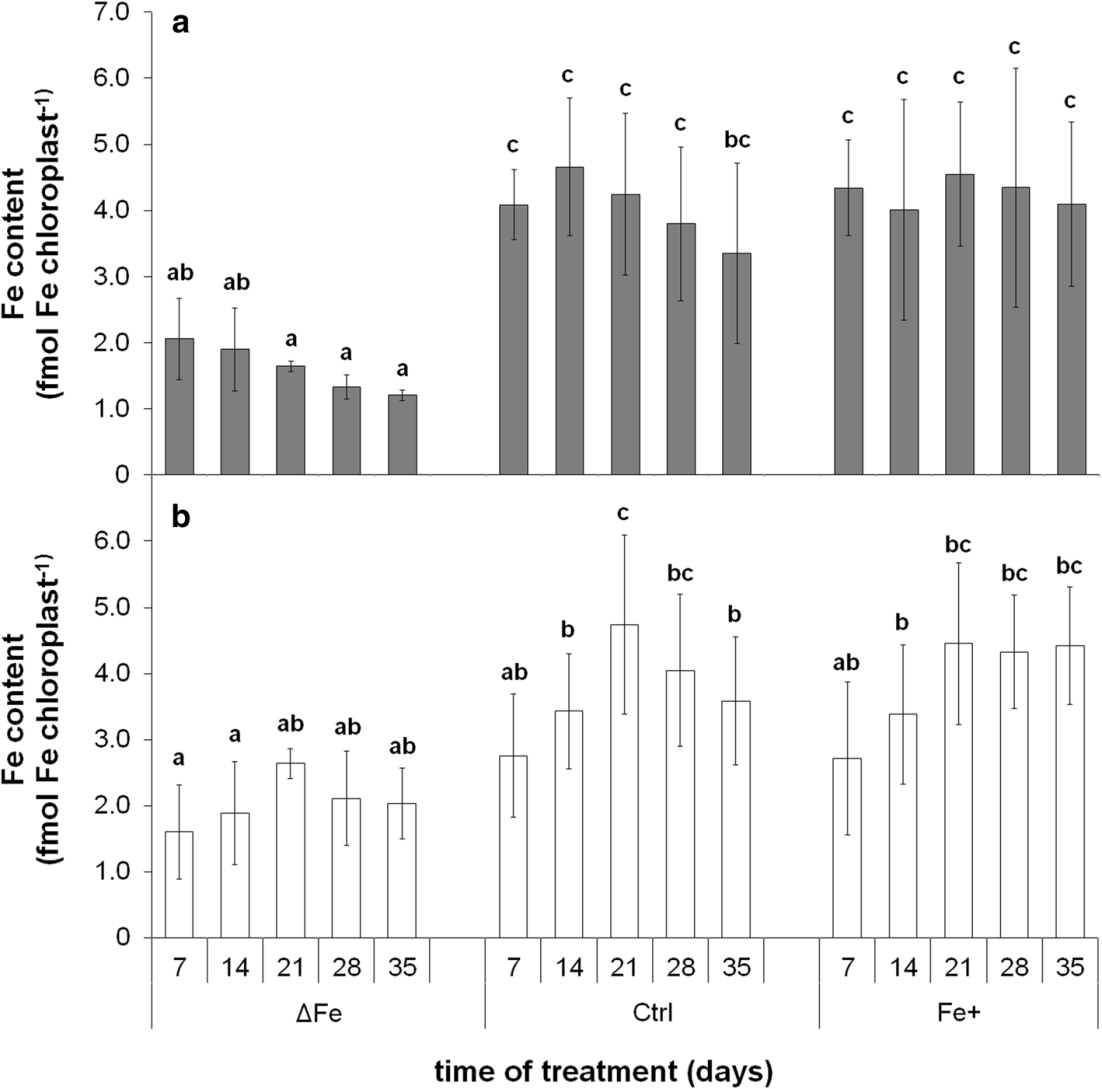
Changes in the Fe content of chloroplasts isolated from the 4th **(a)** and 6th leaves (**b**) during the time of treatment. Treatments as in Fig. 1. Error bars represent SD values. To compare the differences, one-way ANOVAs were performed with Tukey–Kramer post hoc tests on the treatments (*P* < 0.05; *n* = 3 × 3 [biological × technical])

The Fe content was also determined in isolated chloroplasts (Fig. 2a, b, respectively). Under optimal and supraoptimal Fe nutrition conditions, a significant increase in the chloroplast Fe content of 6th leaves was only found during their development, whereas this trend of increase was less pronounced in the Fe-deficient 6th leaves. At the same time, the increase stopped at the second week of treatment in the better developed 4th leaves. After the leaves reached the fully developed stage and thus a maximum in the Fe content of chloroplasts, a slight trend of decrease was observed under Fe deficiency and optimal Fe nutrition. Nevertheless, the chloroplast Fe content remained stable even in older leaves under supraoptimal Fe nutrition.

**Fig. 3:**
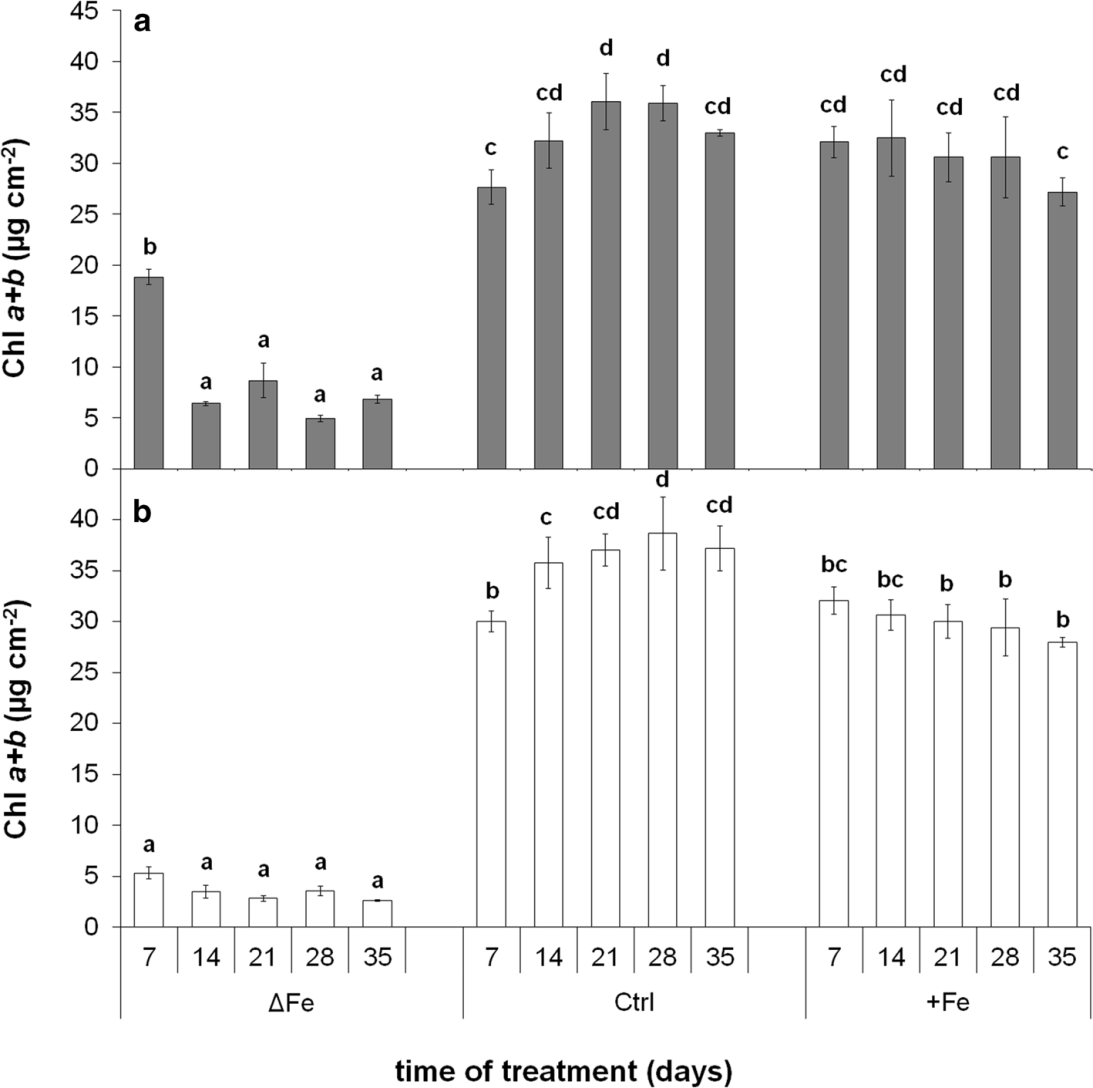
Changes in the Chl *a* + *b* concentration of 4th **(a)** and 6th (**b**) leaves during the time of treatment. Treatments as in Fig. 1. Error bars represent SD values. To compare the differences, one-way ANOVAs were performed with Tukey–Kramer post hoc tests on the treatments (*P* < 0.05; *n* = 3 × 3 [biological × technical])

Chl *a* + *b* concentration of leaves was strongly affected by both the age of leaves and the Fe nutrition status of plants (Fig. 3a, b, respectively). In the leaves of plants grown under optimal Fe nutrition, the Chl concentration rose in both 4th and 6th leaves until it reached the maxima around the 21st day of treatment. Compared to the control leaves, Fe deficiency induced a significant retardation in the accumulation of Chls in both the developing 4th and 6th leaves, whereas the supraoptimal Fe nutrition had hardly any effect on it. Following the full development, a slight but tendentious decrease was found in the Chl concentration in all leaves grown under optimal or supraoptimal Fe nutrition. In contrast, no significant changes were found in the Chl content of Fe-deficient leaves during this time of treatment.

**Fig. 4:**
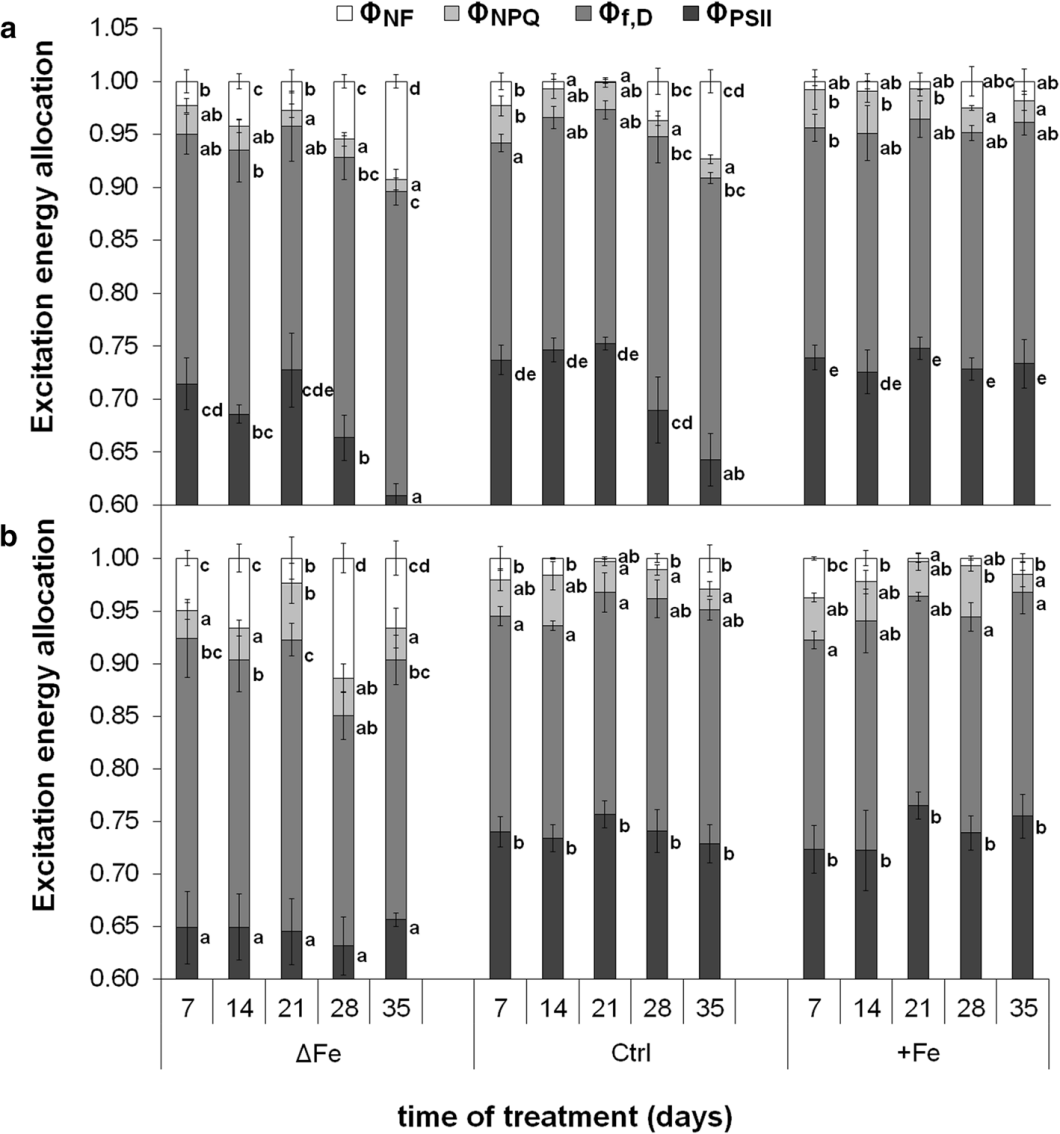
Changes in the excitation energy allocation during the time of treatments in the 4th (**a**) and 6th **(b)** leaves. Treatments as in Fig. 1. Error bars represent SD values. To compare the differences, one-way ANOVAs were performed with Tukey–Kramer post hoc tests on the treatments (*P* < 0.05; *n* = 3 × 3 [biological × technical])

Alteration in the Fe nutrition also led to changes in the excitation energy allocation in leaves (Fig. 4a, b, respectively). Fe deficiency after 28–35 days of treatment decreased the actual photochemical quenching of the PSII reaction centres (*Φ*_PSII_) in the 4th but not in the 6th leaves whereas the excitation energy quenching of the non-functional PSII reaction centres (*Φ*_NF_) increased in both 4th and 6th leaves. Compared to supraoptimal Fe nutrition, *Φ*_NF_ increased more in the 4th than in the 6th leaves under optimal Fe nutrition after 28 and 35 days of treatment in connection with the shading and senescence of the leaves. In contrast, supraoptimal Fe nutrition retained the photochemical activity during the whole time of treatment. No significant changes were found in the non-photochemical quenching of the antennae (*Φ*_NPQ_) and in the fluorescence and constant heat dissipation processes (*Φ*_f,D_) during the treatments.

**Fig. 5:**
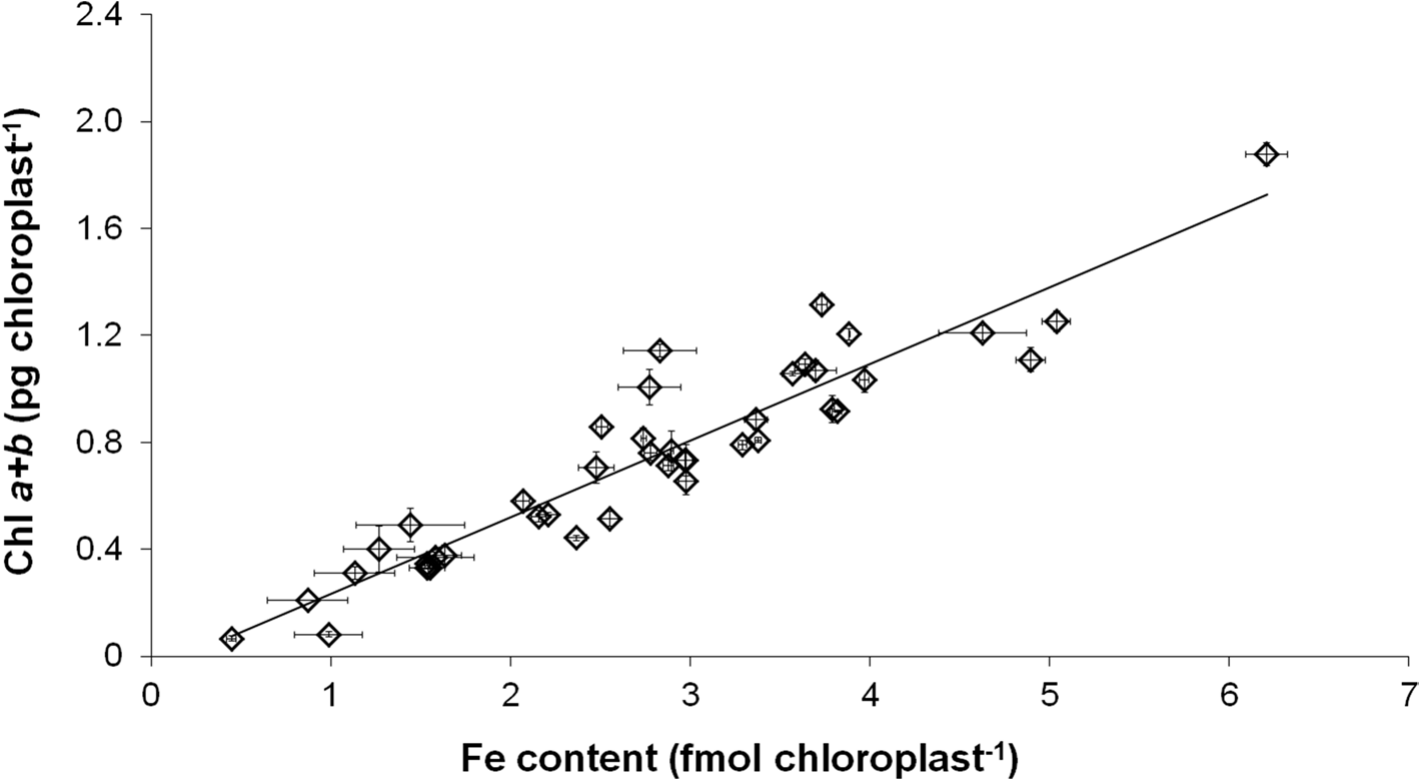
Correlation between Chl *a* + *b* and Fe content of chloroplasts. Linear regression was calculated on 42 averaged individual data pairs where *R*^2^ is 0.8769

**Fig. 6:**
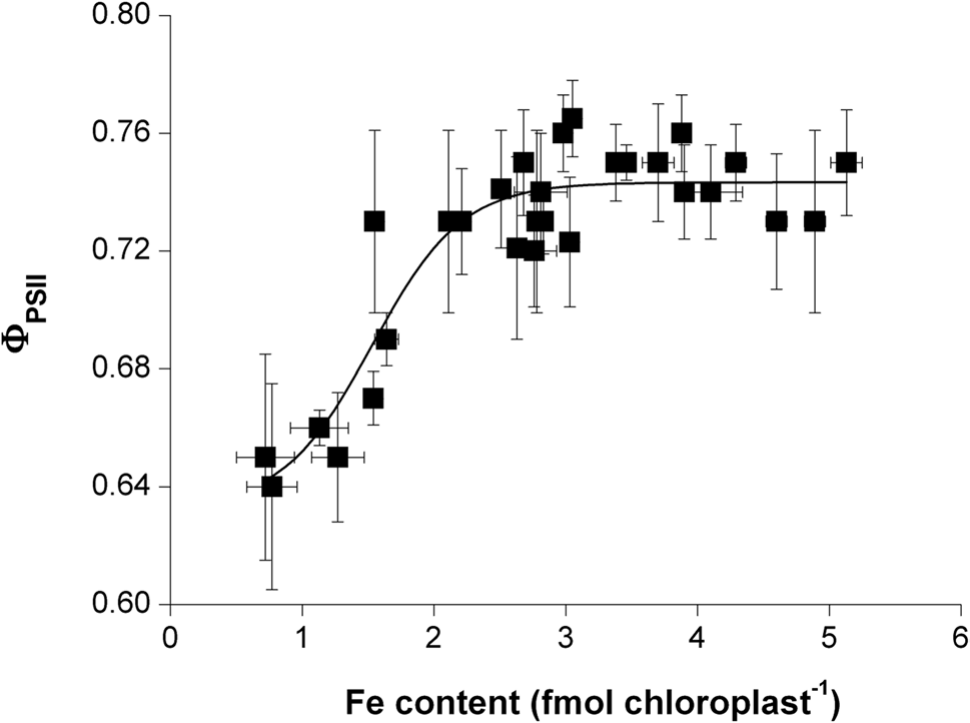
Correlation between the Fe content of chloroplasts and the actual photochemical quenching of PSII reaction centres (*Φ*_PSII_). Boltzmann fit was calculated on 33 individual averaged data pair where *R*^2^ was 0.8455

To connect chloroplast Fe content to the development and status of the photosynthetic apparatus, correlations of these physiological parameters were calculated. A clear linear correlation was found between the Fe and Chl *a* + *b* content of chloroplasts (Fig. 5). Decrease in the chloroplast Fe content also resulted in a significant decrease in the Chl content of chloroplasts independently on the leaf storeys. Changes in Fe nutrition also affected the relation of chloroplast Fe content and the actual quantum efficiency of PSII reaction centres: by the increase in the chloroplast Fe content, *Φ*_PSII_ only increased to a certain level, leading to a saturation-type connection of the two physiological parameters (Fig. 6). The increase in *Φ*_PSII_ saturated at 3.12 ± 0.17 fmol Fe chloroplast^−1^ and at 0.742 ± 0.004 actual quantum efficiency. Further increase in the chloroplast Fe content did not alter the PSII function.

### Time-scale changes in the expression of *BnPIC1*, *BnNiCo* and *MAR1*

**Fig. 7:**
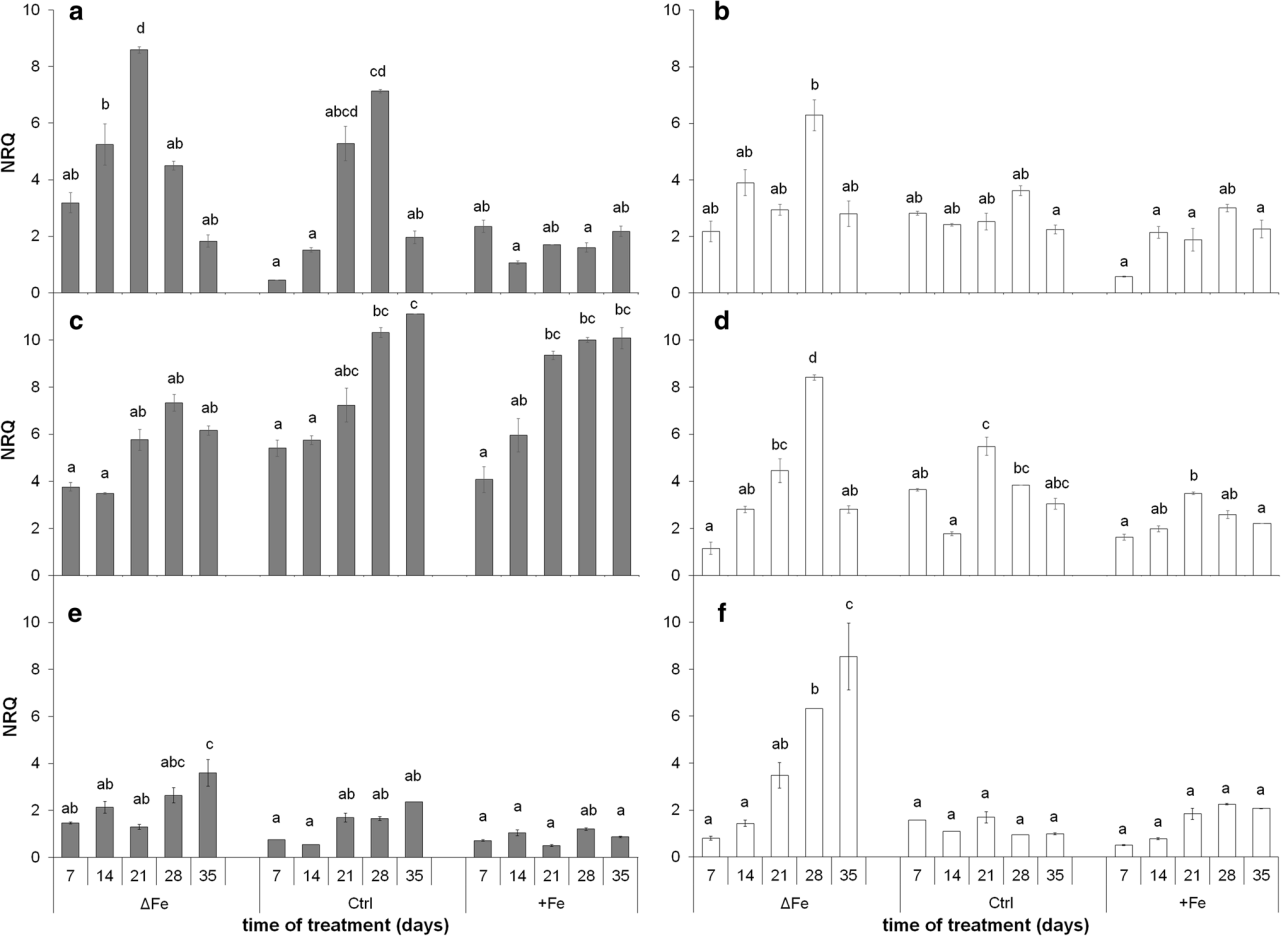
Changes in the transcript levels of *BnPIC1* (**a**, **b**), *BnNiCo* (**c**, **d**) and *BnMAR1* (**e**, **f**). **a**, **c**, **e** 4th leaves. **b**, **d**, **f** 6th leaves. Treatments as in Fig. 1. NRQ, normalized relative quantity of transcript. Error bars represent SE values. To compare the differences, one-way ANOVAs were performed with Tukey–Kramer post hoc tests on the treatments (*P* < 0.05; *n* = 3 × 2 [biological × technical])

The expression of *BnPIC1* was strongly dependent on the Fe nutritional status of the plants—more in 4th and less pronounced but also tendentiously in 6th leaves (Fig. 7a). The lower the amount of available Fe was in the nutrient solution, the higher the relative transcript amount of *BnPIC1* was. The relative transcript amount of *BnPIC1* was also dependent on the developmental status of control and Fe-deficient leaves: in parallel to the area expansion of the leaves, the relative transcript amount also increased, but decreased after reaching the full development (i.e. after 21 days). Interestingly, the peak in the relative transcript amount of *BnPIC1* was found earlier in 4th leaves of Fe-deficient plants than in those of the control indicating an altered developmental pattern of leaves under the limitation of Fe. In contrast, the relative transcript amount did not show these changes in leaves of plants grown under supraoptimal Fe nutrition; its value remained stable during the whole experimental period and similarly low as the values measured at the end of experimental period in leaves of plants grown under Fe-deficient and optimal Fe-nutrition conditions.

Contrary to *BnPIC1*, the relative transcript level of *BnNiCo* showed a positive correlation to the age of leaves (Fig. 7b): although it also increased during the leaf area expansion period, especially in the 4th leaves, it increased further after the stop of the area expansion of leaves. Higher Fe nutrition seemed to enhance the expression. In contrast, the expression was negatively correlated to the Fe nutrition in the 6th leaves: the highest expression was found under Fe deficiency. The relative transcript amount of *BnMAR1* (Fig. 7c) was low and more or less stable under both optimal and supraoptimal Fe nutrition during the whole time of treatment in both leaf storeys (an increase of weak significance was only found in parallel to the ageing of leaves). In leaves of Fe-deficient plants, the relative transcript amount started to increase following the full growth of leaves, whereas *BnMAR1* showed higher induction in the 6th than in the 4th leaves. In Fe-deficient plants, especially in the 6th leaves, the changes in *BnNiCo* and *BnMAR1* showed a positive correlation.

### Changes in the relative amount of NiCo and PIC1 proteins

**Fig. 8:**
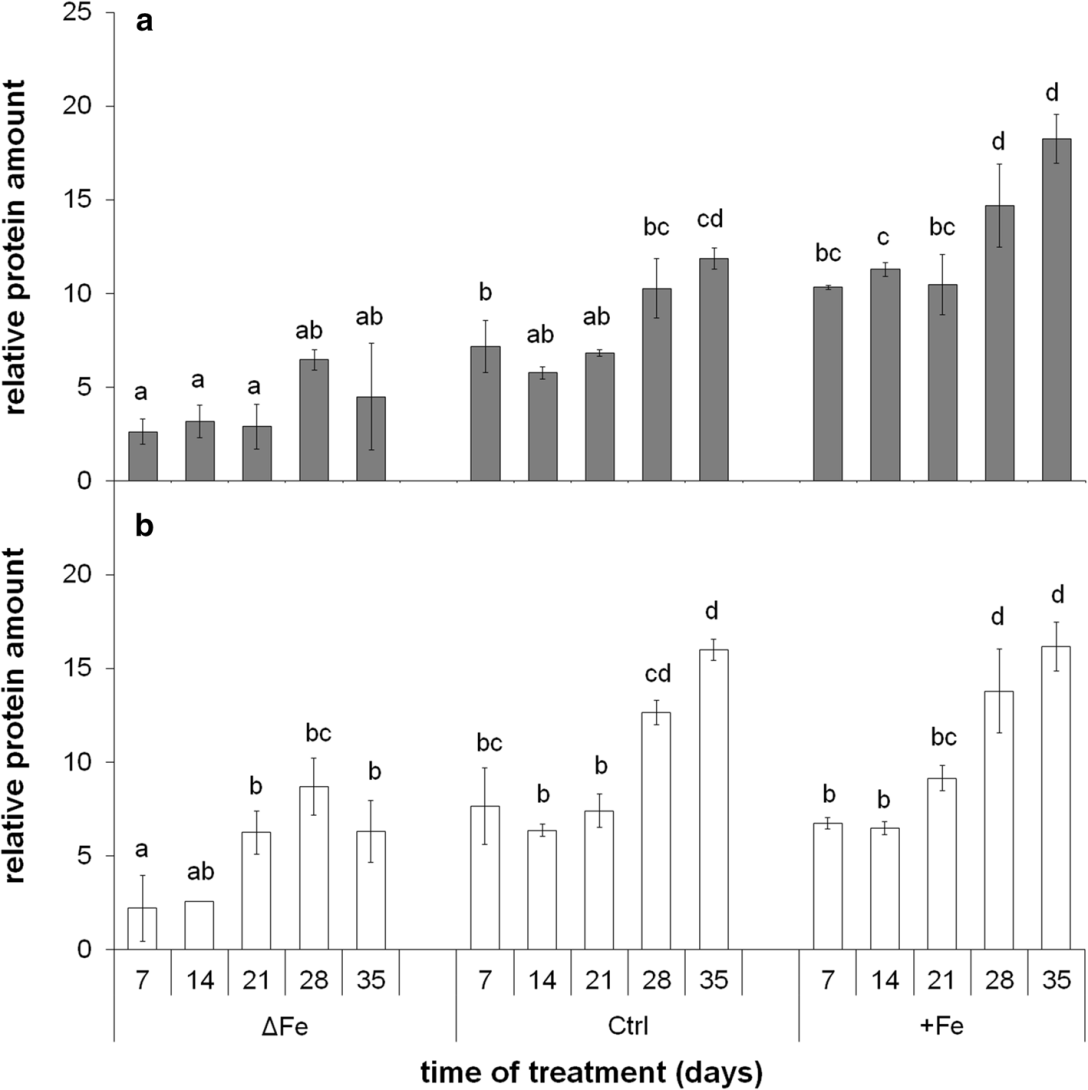
Relative amount of NiCo based on immunoblot analysis. **a** 4th leaves. **b** 6th leaves. Treatments as in Fig. 1. Error bars represent SD values. To compare the differences, one-way ANOVAs were performed with Tukey–Kramer post hoc tests on the treatments (*P* < 0.05; *n* = 3 × 2 [biological × technical])

The NiCo in *Brassica napus* chloroplasts was detected as a protein band of 26 kDa by immunoblots (Suppl. Fig. S3). To validate changes in the transcript amounts of *BnNiCo*, immunoblot assay was performed against BnNiCo protein integrated into chloroplast membranes (Suppl. Fig. S4). Changes in the relative amount of NiCo protein showed good correlation to its transcript amount particularly in the 4th leaves (Fig. 8). Similar to the changes in the expression, the accumulation of NiCo was also dependent on both the age and Fe nutritional status of the leaves. In both 4th and 6th leaves, the amount of NiCo increased in parallel to the development and ageing of leaves, and protein accumulation continued, following the full development of leaves. The accumulation was also dependent on the Fe nutritional status: the more Fe the plants received, the higher accumulation of NiCo was found, especially in older leaves.

The 21 kDa band of PIC1 (TIC21) protein was detected by immunoblots (Suppl. Fig. S5). PIC1 was detected through all the time of treatment in both three treatments (Suppl. Fig. S6). The relative protein amount of PIC1 also correlated to the expression of *BnPIC1* (Suppl. Fig. S7, comparing to Fig. 7a). Thus, in parallel to the increase in the iron nutrition, the relative amount of PIC1 decreased in chloroplasts, whereas it increased during the leaf development especially in Fe-deficient leaves.

### Chloroplast Fe content is related to the transcription of chloroplast Fe transport components

To find general trends in the expression of the genes of interest, their expression was compared to the changes in chloroplast Fe contents during and following the development of leaves (between the 7th and 21st and 21st and 35th days of treatment; Suppl. Fig. S8a and b, respectively) based on the data of the 4th leaves, which showed a clear ageing pattern. During leaf development, a gradual increase in the transcript levels of genes of interest and the Fe content of chloroplasts was found in all treatments. However, the transcript level of *BnPIC1* elevated markedly during leaf development except under supraoptimal Fe nutrition, and together with that of *BnMAR1* were the highest under Fe starvation. At the same time, the expression of *BnNiCo* increased more slowly during leaf development, and reached high transcript levels only in mature leaves in all three treatments.

The 4th leaves slowly turned into senescence following the 21st day of treatment when both Chl and Fe contents decreased together with the inactivation of the photosynthetic apparatus. In parallel, a gradual reduction was found in the expression of *BnPIC1* under Fe-deficient and optimal Fe nutrition. Nevertheless, low expression of *BnPIC1* found during the development was maintained under supraoptimal Fe nutrition during this period, together with only a slight decrease in the Fe content of chloroplasts. In contrast, the expression of *BnMAR1* gradually increased under Fe deficiency in this period, but remained stable under optimal and supraoptimal Fe nutrition. The expression of *BnNiCo* was stable in Fe-deficient leaves, but increased and remained high under optimal and supraoptimal Fe nutrition while Fe content of chloroplasts gradually decreased moderately and slightly, respectively.

## Discussion

The metabolic processes of mesophyll cells are strongly dependent on the availability of Fe. Limitations in the Fe nutrition reduce the Fe content of chloroplasts that also leads to a decrease in the photosynthetic performance and thus to that of the biomass production. Since supraoptimal Fe nutrition is a less severe agronomical problem in comparison with Fe deficiency, we are in a lack of data how saturated Fe nutrition alters the Fe metabolism of mesophyll cells and chloroplasts. The supraoptimal Fe nutrition is a good model system to study the Fe homeostasis in shoot tissues that saturates all Fe-limited processes. Fivefold Fe nutrition was previously shown to induce no physiological alteration in poplar plants (Solti et al. 2008). In the present study, we neither observed Fe accumulation in the chloroplasts, nor any retardation in physiological activity under supraoptimal Fe nutrition; thus, fivefold Fe nutrition has no toxic effects for the applied *Brassica* model. Taxa of the genus *Brassica* are known to tolerate heavy metals in higher concentrations. In *Brassica rapa* (a parental species of *Brassica napus*), the secondary metabolism was shown to be enhanced under slight excess of Fe (50–100 µM) synthesizing amino acids, phenolics and other sulphur-containing metabolites (Jahangir et al. 2010). The vacuolar Fe transporter BnMEB2, also present in roots, contributes to the enhancement of Fe tolerance in *Brassica napus* (Zhu et al. 2016). Accumulation of metabolites and sequestration of Fe into the vacuoles contribute to the detoxification of Fe. Genes involved in the antioxidative defence were also reported to be inducible by the excess of Fe (Vansuyt et al. 1997). The lack of increase in the chloroplast Fe content (Fig. 5) and only slight increase in the leaf Fe content (Fig. 4) under supraoptimal Fe nutrition indicates that Fe retention in the roots may have contributed to the avoidance of Fe toxicity (Wheeler et al. 1985; Becker and Asch 2005; Sperotto et al. 2010; Pinto et al. 2015). In contrast, Fe deficiency inhibited the accumulation of Fe in the leaves and chloroplasts, only a slight increase was measured in the Fe content of 6th leaves during the leaf area expansion. Since in our experiments the Fe-deficiency treatment was preceded by a pre-cultivation in Fe-containing nutrient solution, the source of Fe was the redistribution of Fe within the plants.

Concerning the regulation of Fe homeostasis, a large amount of information is available for roots, but only pieces of information can be found for leaf cells and its organelles (Vigani et al. 2013; Curie and Mari 2017; Hindt et al. 2017). Fe transporters play essential roles in the Fe homeostasis of organelles, including chloroplasts. PIC1 and NiCo might function together in plastid Fe transport (Vigani et al. 2019). Although *PIC1* itself is thought to have a constant expression in all tissues and transcript levels have not been described to depend on Fe deficiency (Duy et al. 2007; Gong et al. 2015), in our study, the expression of both *PIC1* and *NiCo* proved to be dependent on the developmental stage of leaves and the Fe nutrition status of *Brassica napus* plants (Fig. 7a, b, respectively). In the initial stage of leaf development, the expression of both *BnPIC1* and *BnNiCo* was lower than at reaching the full maturity, supporting the hypothesis of a Fe-uptake bypass pathway should exist in chloroplasts of young leaves (López-Millán et al. 2016). The transcript and protein amount of BnPIC1 showed a correlation; thus, we assume that its regulation at the expression level is predominant. The expression of *BnPIC1* was the highest in leaves of Fe-deficient plants that reached leaf maturity, and the lowest in leaves of plants receiving supraoptimal Fe nutrition. The suppression of *PIC1* expression under slight Fe excess implicates that chloroplasts do not contribute to the storage of Fe under physiological conditions (no ferritin was detected under saturation or slight Fe excess nutrition; H–D Pham and A Solti, unpublished results). Rather a feedback down-regulation of the Fe uptake of chloroplasts takes place when a certain level of Fe content under non-toxic conditions has been reached (Solti et al. 2012). In *PIC1*-overexpressing *Arabidopsis*, but also in *Yellow Stripe-like 4/6* mutant lines, Fe was shown to accumulate under high Fe nutrition in chloroplasts (Duy et al. 2007; Divol et al. 2013; Briat et al. 2015). The similar trend of changes of *BnPIC1* and the Fe content of chloroplasts, especially in Fe-deficient plants following the full development and the decreasing *BnPIC1* transcript level at leaf maturity, in parallel to the increased Fe nutrition of the plants suggests that *BnPIC1* is strongly connected to the Fe homeostasis of leaves. Chloroplast-born retrograde signals may have an important role in the regulation of the cellular Fe homeostasis (Vigani et al. 2013; Gong et al. 2015). Surplus Fe in the chloroplasts is known to induce NO production (Touraine et al. 2012). In contrast, S-nitrosoglutathione reductase contributes to the elimination of S-nitrosoglutathione and thus nitrosylation processes (Kolbert et al. 2019). Under optimal iron nutrition, the overexpression of S-nitrosoglutathione reductase increased the expression of *ferritin 2*, *PIC1*, *VIT*, but decreased that of *Ferric Chelate Reductase 7* in young leaves, indeed, decreased all of that in mature leaves (Wen et al. 2019). Since NO is known to be produced under the excess of Fe in chloroplasts, the results of Wen et al. (2019) seem to correspond to our measurements in plants of supraoptimal Fe nutrition. In *Arabidopsis thaliana*, NEET was very recently also pointed out as a possible key regulator of Fe metabolism in chloroplasts. Disruption in the function of NEET results in an uncoupling in the transfer of chloroplast-synthetized Fe_2_S_2_ cluster to cytosolic proteins as well as in an accumulation of Fe in chloroplasts (Zandalinas et al. 2019). In our measurements, limited Fe accumulation in chloroplasts initially induced the Fe uptake machinery, but reaching leaf maturity acted as a repressor of *BnPIC1* transcription. In parallel, decrease in the chloroplast Fe content and inactivation of PSII reaction centres together reflect the induction of senescence processes after reaching the maturity stage of leaves. Iron deficiency in the leaves induces senescence processes (Wang et al. 2016). Under Fe-deficient nutrition, a tendency of decay in the photosynthetic apparatus (decrease in Φ_PSII_ and Chl content, increase in Φ_NF_) was measured as a signal for senescence. In parallel to the changes in the photosynthetic functions, chloroplast Fe content of plants decreased slightly. These slight changes were also measured, yet less markedly in plants grown under optimal Fe nutrition. In comparison to plants grown under optimal Fe nutrition, leaves of Fe-deficient plants showed the responses in the photosynthetic apparatus earlier. Nutrient deprivation enhances the remobilization and reorganizing of nutrients in *Brassica napus* as senescence progresses (Maillard et al. 2015; Pottier et al. 2019; Mari et al. 2020). In contrast, supraoptimal Fe nutrition even more slightly delayed these signs of senescence processes. These findings are in line with the role of PIC1 in chloroplast Fe uptake and confirm the results that the expression of both ferritins and Fe superoxide dismutase decreased in *PIC1*-RNAi down-regulation constructs but increased in *PIC1*-overexpressing lines of tobacco in parallel to the changes in the chloroplast Fe content (Gong et al. 2015). In *Arabidopsis*, however, *PIC1* knockouts exhibited decreased chloroplast Fe superoxide dismutase transcripts and protein but strongly increased ferritin RNA and proteins (Duy et al. 2007). If the latter discrepancy is due to different regulation of ferritin expression in tobacco and *Arabidopsis* or an effect of knockdown (tobacco *PIC1*) versus knockout (*Arabidopsis PIC1*) still has to be clarified.

MAR1 (IREG3; a sequence homologue of mouse IREG1, Fe efflux mediator in epithelial cells) is suggested to be involved in Fe import from the cytosol (Yang et al. 2010), possibly translocating Fe–nicotianamine complexes. Since the expression of *BnMAR1* only increased in older leaves of Fe-deficient plants (Fig. 7c), together with the unchanged chloroplast Fe content in this stage, its contribution to the Fe uptake of chloroplasts is debated.

Concerning NiCo, its relative transcript and protein amounts also correlated; thus, the expression level regulation of this component seems to be predominant, too. Similar to *BnPIC1*, the expression of *BnNiCo* also increased in all leaf storeys and treatments during leaf development. In contrast to *BnPIC1*, however, the relative transcript and protein amounts of *BnNiCo* did not increase by Fe deficiency. Both the transcript and protein amount of BnNiCo showed a trend of increase even after leaves had reached their full development. Thus, PIC1 and NiCo are assumed to stay under independent regulation. These results question whether these components contribute in the chloroplast Fe transportation in the same action. Taken together the changes in the chloroplast Fe content and relative transcript and protein amounts of NiCo, it seems unsupported that NiCo would only contribute to the Fe acquisition of chloroplasts, but may also be involved in either Fe sensing or Fe release from chloroplasts. Nevertheless, parallel decrease in the expression of *BnPIC1* and the chloroplast Fe content indicates that PIC1 may not contribute to this Fe release from chloroplasts. Under Fe deficiency, the increasing expression of *BnMAR1* following the full development of leaves together with the parallel decrease in the Fe content of chloroplasts and inactivation of PSII reaction centres are tempting to speculate that MAR1 is not involved in the Fe acquisition of chloroplasts but rather in the Fe release from the plastids and the redistribution of Fe in the cells. Since under Fe deficiency, the expression of *TAP1*, a hypothetical chloroplast Fe–S cluster exporter, was previously shown to be upregulated in parallel to *MAR1* (Yang et al. 2010), remodelling of Fe content not only covers the export of Fe (complexes) but also Fe-containing cofactors. On the other hand, neither the overexpression of *H89C* or *NEET*, having profound roles in the Fe_2_S_2_ cluster incorporation and Fe sensing in chloroplasts, induced any significant changes in the expression of neither *MAR1*, nor *PIC1* (Zandalinas et al. 2019). In addition, based on an autophagy mutant *atg5-1* where decomposing of chloroplasts is affected, retranslocation of Fe strongly decreases from vegetative tissues (Mari et al. 2020). Fe homeostasis shifts upon developmental senescence, whereas Fe shortage is documented to induce premature senescence (Pottier et al. 2019). In senescing *Arabidopsis* leaves, specific up-regulation of vacuolar Fe transporter NRAMP3 has been found (Pottier et al. 2014). The MATE transporter ELS1 was shown to be involved in leaf senescence and Fe remobilisation in *Arabidopsis*. Since its close orthologues are citrate transporters that are important in xylem citrate loading and thus the xylem mobility of Fe (Wang et al. 2016), a xylem transport of Fe during senescence may be expected. Although Fe nutritional status seems to contribute to the regulation of chloroplast Fe remodelling and in a broader context, the remobilisation of Fe, the signalling of the operation of chloroplast Fe transport machinery needs further investigations.

In conclusion, the developmental status of leaves and the Fe nutrition status of the plants have a strong regulatory role on the Fe homeostasis of chloroplasts. The Fe uptake transporter PIC1 is upregulated by Fe starvation and in developing leaves but downregulated after leaves reached the full development and possibly upon senescence signals. NiCo, supposed to be an interaction partner of PIC1, seems to be regulated and operating distinctly. Saturation of the Fe demand of chloroplasts seems to be linked to the delay of senescence of chloroplasts that also lead to keep chloroplast Fe homeostasis and Fe transport machinery in a rather constant status, whereas Fe deficiency induces a remodelling in the Fe content of cells following the development of the photosynthetic apparatus in the chloroplasts.

### *Author contribution statement*

ÁS designed and supervised the study. Physiological and chloroplast Fe content measurements were performed by H-DP, BM, and FF. KS, BB and FB designed and tested the primers for reference genes. H-DP, SP, MS-K and LT identified *AtPIC1*, *AtMAR1* and *AtNiCo* orthologues in the *Brassica* genome and performed the expression analysis studies. Antibody against NiCo was constructed by KP, immunoblot studies were performed by H-DP and ÉS. ÁS and H-DP wrote and all authors critically reviewed the manuscript.

## Electronic supplementary material

Below is the link to the electronic supplementary material.

Supplementary file1 (PDF 686 kb)

## Abbreviations

IREG: Iron regulated protein
MAR: Multiple antibiotic resistance protein
NiCo: Nickel–cobalt transporter
PIC: Permease in chloroplast

## References

[CR1] Andersen CL, Jensen JL, Ørntoft TF (2004). Normalization of real-time quantitative reverse transcription-PCR data: a model-based variance estimation approach to identify genes suited for normalization, applied to bladder and colon cancer data sets. Cancer Res.

[CR2] van Bambeke F, Balzi E, Tulkens PM (2000). Antibiotic efflux pumps. Biochem Pharmacol.

[CR3] Basa B, Lattanzio G, Solti Á, Tóth B, Abadía J, Fodor F, Sárvári É (2014). Changes induced by cadmium stress and iron deficiency in the composition and organization of thylakoid complexes in sugar beet (*Beta vulgaris* L.). Environ Exp Bot.

[CR4] Becker M, Asch F (2005). Fe toxicity in rice—conditions and management concepts. J Plant Nutr Soil Sci.

[CR5] Briat JF, Lobréaux S (1997). Fe transport and storage in plants. Trends Plant Sci.

[CR6] Briat JF, Ravet K, Arnaud N, Duc C, Boucherez J, Touraine B, Cellier F, Gaymard F (2010). New insights into ferritin synthesis and function highlight a link between Fe homeostasis and oxidative stress in plants. Ann Bot.

[CR7] Briat JF, Rouached H, Tissot N, Gaymard F, Dubos C (2015). Integration of P, S, Fe and Zn nutrition signals in *Arabidopsis thaliana* potential involvement of phosphate starvation response 1 (PHR1). Front Plant Sci.

[CR8] Castagna A, Donnini S, Ranieri A, Ozturk M, Athar HR, Ashraf M (2009). Adaptation to iron-deficiency requires remodelling of plant metabolism: an insight in chloroplast biochemistry and functionality. Salinity and water stress.

[CR9] Conte SS, Lloyd AM (2010). The MAR1 transporter is an opportunistic entry point for antibiotics. Plant Signal Behav.

[CR10] Conte S, Stevenson D, Furner I, Lloyd A (2009). Multiple antibiotic resistance in *Arabidopsis thaliana* is conferred by mutations in a chloroplast-localized transport protein. Plant Physiol.

[CR11] Curie C, Cassin G, Couch D, Divol F, Higuchi K, Le Jean M, Misson J, Schikora A, Czemic P, Mari S (2009). Metal movement within the plant: contribution of nicotianamine and yellow stripe 1-like transporters. Ann Bot.

[CR12] Curie C, Mari S (2017). New routes for plant iron mining. New Phytol.

[CR13] Czechowski T, Stitt M, Altmann T, Udvardi MK, Scheible WR (2005). Genome wide identification and testing of superior reference genes for transcript normalization in *Arabidopsis*. Plant Physiol.

[CR14] Divol F, Couch D, Conéjéro G, Roschzttardtz H, Mari S, Curie C (2013). The *Arabidopsis* YELLOW STRIPE LIKE 4 and 6 transporters control Fe release from the chloroplast. Plant Cell.

[CR15] Duy D, Stübe R, Wanner G, Philippar K (2011). The chloroplast permease PIC1 regulates plant growth and development by directing homeostasis and transport of iron. Plant Physiol.

[CR16] Duy D, Wanner G, Meda A, von Wiren N, Soll J, Philippar K (2007). PIC1 an ancient permease in *Arabidopsis chloroplasts* mediates Fe transport. Plant Cell.

[CR17] Eitinger T, Suhr J, Moore L, Smith JA (2005). Secondary transporters for nickel and cobalt ions: theme and variations. Biometals.

[CR18] Filiz E, Akbudak MA (2019). Investigation of PIC1 (permease in chloroplasts 1) gene’s role in iron homeostasis: bioinformatics and expression analyses in tomato and sorghum. Biometals.

[CR19] Gong X, Guo C, Terachi T, Cai H, Yu D (2015). Tobacco PIC1 mediates Fe transport and regulates chloroplast development. Plant Mol Biol Rep.

[CR20] Halliwell B, Gutteridge JMC (1992). Biologically relevant metal ion-dependent hydroxyl radical generation: an update. FEBS Lett.

[CR21] Hantzis LJ, Kroh GE, Jahn CE, Cantrell M, Peers G, Pilon M, Ravet K (2018). A program for iron economy during deficiency targets specific Fe proteins. Plant Physiol.

[CR22] Hendrickson L, Förster B, Pogson BJ, Chow WS (2005). A simple chlorophyll fluorescence parameter that correlates with the rate coefficient of photoinactivation of photosystem II. Photosynth Res.

[CR23] Hindt MN, Akmakjian GZ, Pivarski KL, Punshon T, Baxter I, Salt DE, Guerinot ML (2017). BRUTUS and its paralogs BTS LIKE1 and BTS LIKE2 encode important negative regulators of the iron deficiency response in *Arabidopsis thaliana*. Metallomics.

[CR24] Jahangir M, Abdel-Farid IB, Choi YH, Verpoorte R (2008). Metal ion-inducing metabolite accumulation in *Brassica rapa*. J Plant Physiol.

[CR25] Jeong J, Cohu C, Kerkeb L, Pilon M, Connolly EL, Guerinot ML (2008). Chloroplast Fe (III) chelate reductase activity is essential for seedling viability under iron limiting conditions. Proc Natl Acad Sci USA.

[CR26] Jeong J, Guerinot ML (2009). Homing in on Fe homeostasis in plants. Trends Plant Sci.

[CR27] Kobayashi T, Nishizawa NK (2012). Iron uptake translocation and regulation in higher plants. Annu Rev Plant Biol.

[CR28] Kolbert Z, Feigl G, Freschi L, Poór P (2019). Gasotransmitters in action: Nitric oxide-ethylene crosstalk during plant growth and abiotic stress responses. Antioxidants.

[CR29] Kovács-Bogdán E, Soll J, Bölter B (2010). Protein import into chloroplasts: the Tic complex and its regulation. Biochim Biophys Acta Mol Cell Res.

[CR30] Laemmli UK (1970). Cleavage of structural proteins during assembly of the head of bacteriophage T4. Nature.

[CR31] López-Millán AF, Duy D, Philippar K (2016). Chloroplast Fe transport proteins—function and impact on plant physiology. Front Plant Sci.

[CR32] Maillard A, Diquélou S, Billard V, Laîné P, Garnica M, Prudent M, Garcia-Mina JM, Yvin JC, Ourry A (2015). Leaf mineral nutrient remobilization during leaf senescence and modulation by nutrient deficiency. Front Plant Sci.

[CR33] Müller B, Kovács K, Pham H-D, Kavak Y, Pechoušek J, Machala L, Zboril R, Szenthe K, Abadía J, Fodor F, Klencsár Z, Solti Á (2019). Chloroplasts preferentially take up ferric-citrate over iron-nicotianamine complexes in *Brassica napus*. Planta.

[CR34] Muneer S, Ahmad J, Bashir H, Moiz S, Qureshi MI (2014). Studies to reveal importance of Fe for Cd tolerance in *Brassica juncea*. Int J App Biotech Biochem.

[CR35] Pfaffl MW (2001). A new mathematical model for relative quantification in real-time RT–PCR. Nucleic Acids Res.

[CR36] Pinto SDS, Souza AED, Oliva MA, Pereira EG (2015). Oxidative damage and photosynthetic impairment in tropical rice cultivars upon exposure to excess iron. Sci Agric.

[CR37] Pottier M, Masclaux-Daubresse C, Yoshimoto K, Thomine S (2014). Autophagy as a possible mechanism for micronutrient remobilization from leaves to seeds. Front Plant Sci.

[CR38] Pottier M, Dumont J, Masclaux-Daubresse C, Thomine S (2019). Autophagy is essential for optimal translocation of iron to seeds in *Arabidopsis*. J Exp Bot.

[CR39] Porra RJ, Thompson WA, Kriedemann PE (1989). Determination of accurate extinction coefficients and simultaneous equations for assaying chlorophylls a and b extracted with four different solvents: verification of the concentration of chlorophyll standards by atomic absorption spectroscopy. Biochim Biophys Acta.

[CR40] Ravet K, Touraine B, Boucherez J, Briat JF, Gaymard F, Cellier F (2009). Ferritins control interaction between Fe homeostasis and oxidative stress in *Arabidopsis*. Plant J.

[CR41] Richardson LG, Schnell DJ (2019). Origins, function, and regulation of the TOC–TIC general protein import machinery of plastids. J Exp Bot.

[CR42] Roschzttardtz H, Conéjéro G, Divol F, Alcon C, Verdeil JL, Curie C, Mari S (2013). New insights into Fe localization in plant tissues. Front Plant Sci.

[CR43] Rout GR, Sahoo S (2015). Role of iron in plant growth and metabolism. Rev Agricult Sci.

[CR44] Sárvári É, Solti Á, Basa B, Mészáros I, Lévai L, Fodor F (2011). Impact of moderate Fe excess under Cd stress on the photosynthetic performance of poplar (*Populus jacquemontiana* var. *glauca* cv. Kopeczkii). Plant Physiol Biochem.

[CR45] Schwacke R, Schneider A, van der Graaff E, Fischer K, Catoni E, Desimone M, Frommer WB, Flugge UI, Kunze R (2003). ARAMEMNON a novel database for *Arabidopsis* integral membrane proteins. Plant Physiol.

[CR46] Shi LX, Theg SM (2013). The chloroplast protein import system: from algae to trees. Biochim Biophys Acta Mol Cell Res.

[CR47] Shimoni-Shor E, Hassidim M, Yuval-Naeh N, Keren N (2010). Disruption of Nap14 a plastid-localized non-intrinsic ABC protein in *Arabidopsis thaliana* results in the over-accumulation of transition metals and in aberrant chloroplast structures. Plant Cell Environ.

[CR48] Smith GF, McCurdy WH, Diehl H (1952). The colorimetric determination of Fe in raw and treated municipal water supplies by use of 4:7-diphenyl-1:10-phenanthroline. Analyst.

[CR49] Solti Á, Kovács K, Basa B, Vértes A, Sárvári É, Fodor F (2012). Uptake and incorporation of iron in sugar beet chloroplasts. Plant Physiol Biochem.

[CR50] Solti Á, Müller B, Czech V, Sárvári É, Fodor F (2014). Functional characterization of the chloroplast ferric chelate oxidoreductase enzyme. New Phytol.

[CR51] Solti Á, Kovács K, Müller B, Vázquez S, Hamar É, Pham HD, Tóth B, Abadía J, Fodor F (2016). Does a voltage-sensitive outer envelope transport mechanism contributes to the chloroplast iron uptake?. Planta.

[CR52] Sperotto RA, Ricachenevsky FK, Stein RJ, Waldow VA, Fett JP (2010). Iron stress in plants: dealing with deprivation and overload. Plant Stress.

[CR53] Tarantino D, Morandini P, Ramirez L, Soave C, Murgia I (2011). Identification of an *Arabidopsis* mitoferrin-like carrier protein involved in Fe metabolism. Plant Physiol Biochem.

[CR54] Teng YS, Su YS, Chen LJ, Lee YJ, Hwang I, Li HM (2006). Tic21 is an essential translocon component for protein translocation across the chloroplast inner envelope membrane. Plant Cell.

[CR55] Terry N, Abadía J (1986). Function of iron in chloroplasts. J Plant Nutr.

[CR56] Touraine B, Briat JF, Gaymard F (2012). GSH threshold requirement for NO-mediated expression of the *Arabidopsis* AtFer1 ferritin gene in response to iron. FEBS Lett.

[CR57] Vansuyt G, López F, Inzé D, Briat JF, Fourcroy P (1997). Iron triggers a rapid induction of ascorbate peroxidase gene expression in *Brassica napus*. FEBS Lett.

[CR58] Voith von Voithenberg L, Park J, Stübe R, Lee Y, Philippar K (2019). A novel ABCI multisubunit ECF/ABC transporter of prokaryotic origin in chloroplast metal homeostasis. Front Plant Sci.

[CR59] Vigani G, Zocchi G, Bashir K, Philippar K, Briat J (2013). Signals from chloroplasts and mitochondria for Fe homeostasis regulation. Trends Plant Sci.

[CR60] Vigani G, Hanikenne M, Logan DC (2018). Metal homeostasis in plant mitochondria. Annual plant reviews 50: plant mitochondria.

[CR61] Vigani G, Solti Á, Thomine S, Philippar K (2019). Essential and detrimental—an update on intracellular iron trafficking and homeostasis. Plant Cell Physiol.

[CR62] Wang Z, Qian C, Guo X, Liu E, Mao K, Mu C, Chen N, Zhang W, Liu H (2016). ELS1, a novel MATE transporter related to leaf senescence and iron homeostasis in *Arabidopsis thaliana*. Biochem Biophys Res Commun.

[CR63] Waszczak C, Carmody M, Kangasjärvi J (2018). Reactive oxygen species in plant signalling. Annu Rev Plant Biol.

[CR64] Watson SJ, Sowden RG, Jarvis P (2018). Abiotic stress-induced chloroplast proteome remodelling: a mechanistic overview. J Exp Bot.

[CR65] Wen D, Sun S, Yang W, Zhang L, Liu S, Gong B, Shi Q (2019). Overexpression of S-nitrosoglutathione reductase alleviated iron-deficiency stress by regulating iron distribution and redox homeostasis. J Plant Physiol.

[CR66] Wheeler BD, Al-Farraj MM, Red C (1985). Iron toxicity to plans in base-rich wetlands: comparative effects on the distribution and growth of *Epilobium hirsutum* L and *Juncus subnodulosus* Schrank. New Phytol.

[CR67] Wu H, Ji Y, Du J, Kong D, Liang H, Ling HQ (2010). Clpc1 an ATP-dependent Clp protease in plastids is involved in Fe homeostasis in *Arabidopsis* leaves. Ann Bot.

[CR68] Yang TJ, Lin WD, Schmidt W (2010). Transcriptional profiling of the *Arabidopsis* iron deficiency response reveals conserved transition metal homeostasis networks. Plant Physiol.

[CR69] Zandalinas SI, Song L, Sengupta S, McInturf S, Grant DG, Marjault HB, Castro-Guerrero NA, Burks D, Azad RK, Mendoza-Cozatl DG, Nechushtai R, Mittler R (2019). Expression of a dominant-negative AtNEET-H89C protein disrupts iron-sulfur metabolism and iron homeostasis in *Arabidopsis*. Plant J.

[CR70] Zhang JC, Castellano C, Tucker A, Li L, Kaplan J, Guerinot ML, Jeong J, Schmidt W (2018). Characterization of a mitochondrial Ferroportin in *Arabidopsis thaliana*. 19th International symposium on iron nutrition and interactions in plants.

[CR71] Zhu W, Zuo R, Zhou R, Huang J, Tang M, Cheng X, Liu Y, Tong C, Xiang Y, Dong C, Liu S (2016). Vacuolar iron transporter *BnMEB2* is involved in enhancing Fe tolerance of *Brassica napus*. Front Plant Sci.

