# Supplementary material for: The developmental and iron nutritional pattern of PIC1 and NiCo does not support their interdependent and exclusive collaboration in chloroplast iron transport in Brassica napus
Source: Planta. 2020 Apr 15;251(5):96. doi: 10.1007/s00425-020-03388-0 (PMC7214486; doi:10.1007/s00425-020-03388-0)
Supplement: Supplementary file 1 — Supplementary file1 (PDF 686 kb) [file 425_2020_3388_MOESM1_ESM.pdf]

Supplementary Material

The developmental and iron nutritional pattern of PIC1 and NiCo does not support their interdependent and exclusive collaboration in chloroplast iron transport in *Brassica napus*

Hong Diep Pham, Sára Pólya, Brigitta Müller, Kálmán Szenthe, Máté Sági-Kazár, Barbara Bánkúti, Ferenc Bánáti, Éva Sárvári, Ferenc Fodor, László Tamás, Katrin Philippar, Ádám Solti*

***Correspondence:**

Ádám Solti, PhD

adam.solti@ttk.elte.hu

Supplementary Figures

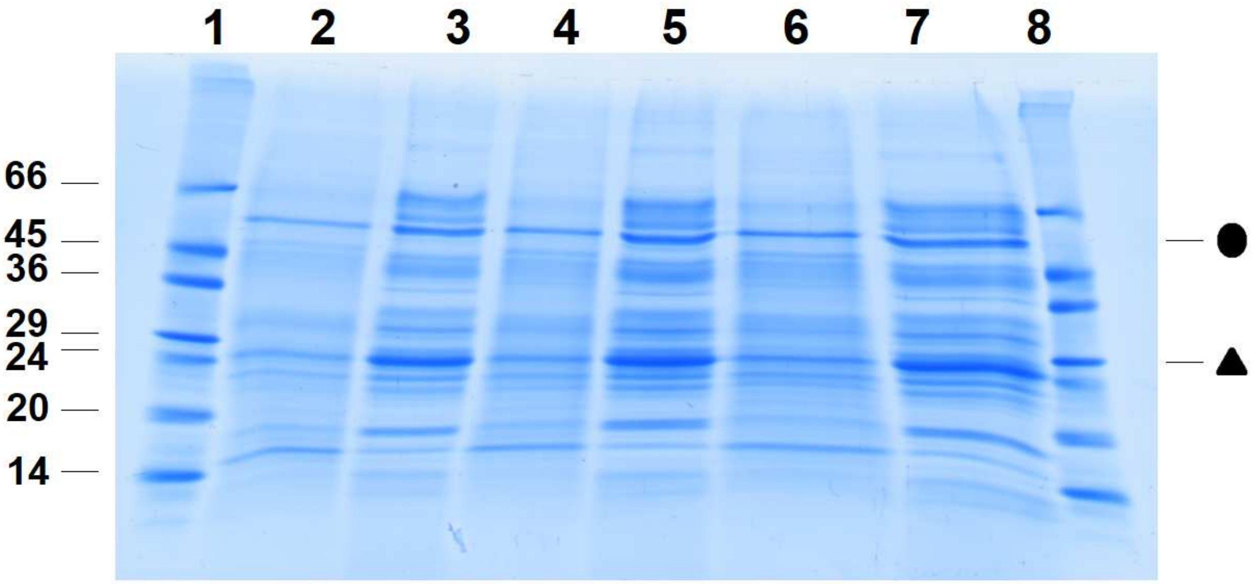

Supplementary Fig. S1 Representative Coomassie stained SDS PAGE pattern of total chloroplast (2; 4; 6) and leaf (3; 5; 7) proteins isolated from 21-days old 4th leaves of plants grown under Fe deficient (2; 3) optimal Fe nutrition (4; 5) and superoptimal Fe nutrition (6; 7) conditions. As for molecular weight standards (1; 8), α-lactalbumin, trypsin inhibitor, trypsinogen, carbonic anhydrase, glycerinaldehyde-3-phosphate dehydrogenase, ovalbumin; bovine serum albumin (Sigma-Aldrich; Lot.: 123H9458; 5 μg protein in total) were used. Lanes were loaded with 18; 12; 15; 8; 11; 8 μg solubilised protein (lanes 2; 3; 4; 5; 6; 7, respectively). Circle – RbcL; triangle – apoLhcII. Ratios of RbcL to apoLhcII were 0.507; 0.580; 0.653; 0.673; 0.586; 0.664 in lanes 2; 3; 4; 5; 6; 7, respectively (the intactness of Fe deficient, optimal Fe nutrition and superoptimal Fe nutrition chloroplast samples were 87.5; 97.1; 88.2%, respectively).

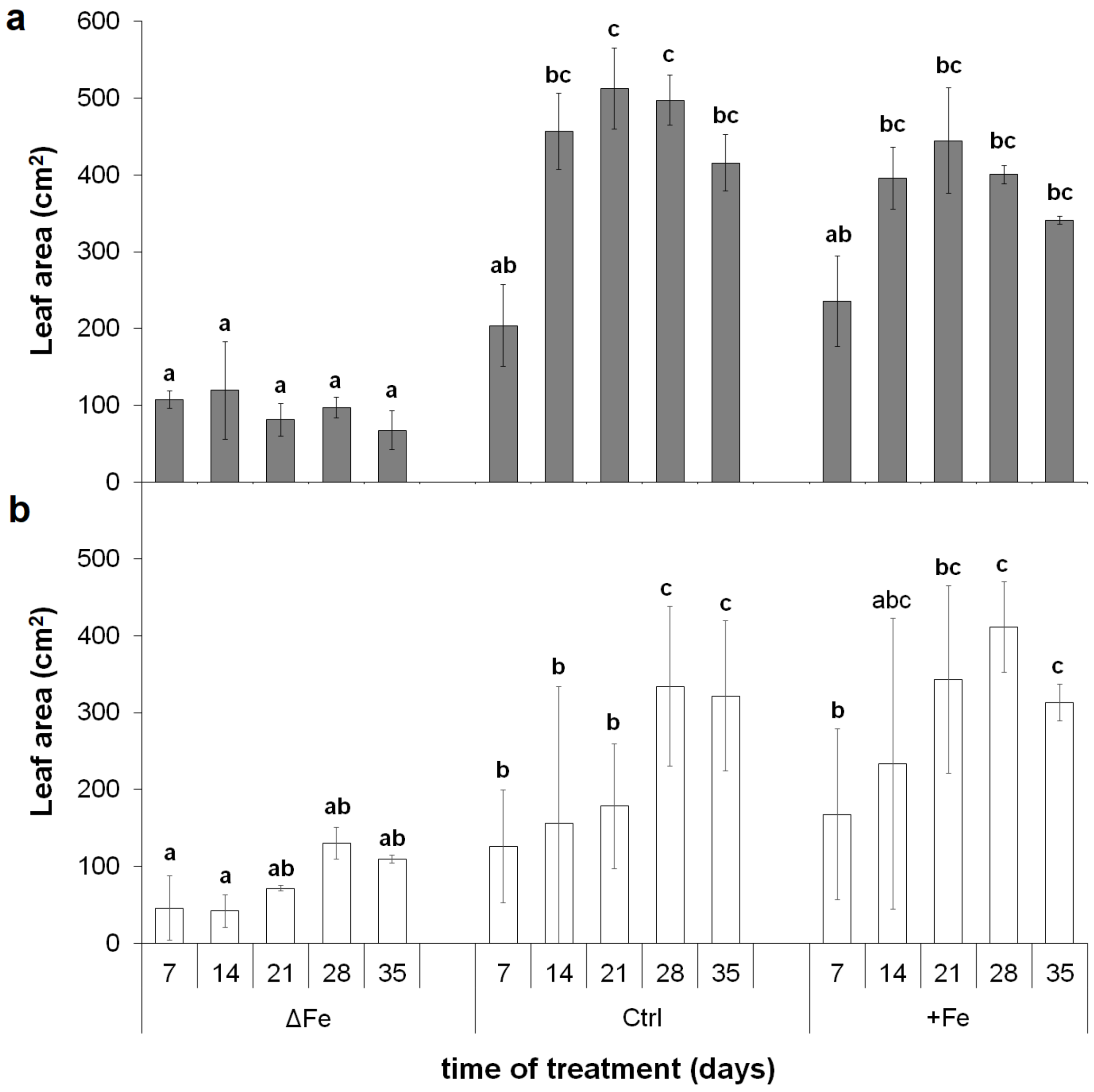

Supplementary Fig. S2 Changes in the leaf area of *Brassica napus* plants during the time of treatment; **a** – 4th leaves; **b** – 6th leaves. ΔFe – Fe deficiency, Ctrl – optimal Fe nutrition (control), +Fe – superoptimal Fe nutrition. Error bars represent SD values. To compare the differences, one-way ANOVAs were performed with Tukey-Kramer *post-hoc* tests on the treatments ($P<0.05$; $n=3\times3$ [biological×technical])

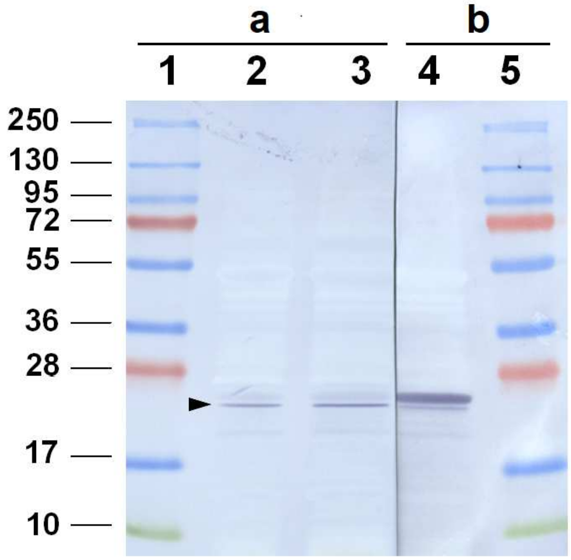

Supplementary Fig. S3 Identification of BnNiCo protein in total chloroplast protein samples isolated from 4th leaves. Marks: 1, 5 – molecular weight standard; 2, 4 – proteins solubilized according to Laemmli UK (1970) Nature 227: 680; 3 – total proteins solubilized according to Duy D et al. (2007) Plant Cell 19: 986. using 250 μg ml^{-1} Pefabloc instead of PMSF. Samples were run on the same gel and blotted to the same nitrocellulose membrane. After blotting, the membrane was cut (**a** and **b**) and treated with the antibodies separately. **a** – immunoblot against NiCo using rabbit polyclonal antiserum against the recombinant, C-terminal part of the *Pisum sativum* NiCo protein (amino acids 236–375); **b** – immunoblot against LHCII using rabbit polyclonal antibodies against Light Harvesting Complex II apoprotein. As for molecular weight standards, PageRuler™ Plus Prestained Protein Ladder (Thermo-Fisher Scientific; Lot.: 00463463) was used. Lanes were loaded with 30 μg solubilised protein. Arrowhead is pointing on BnNiCo at 26 kDa

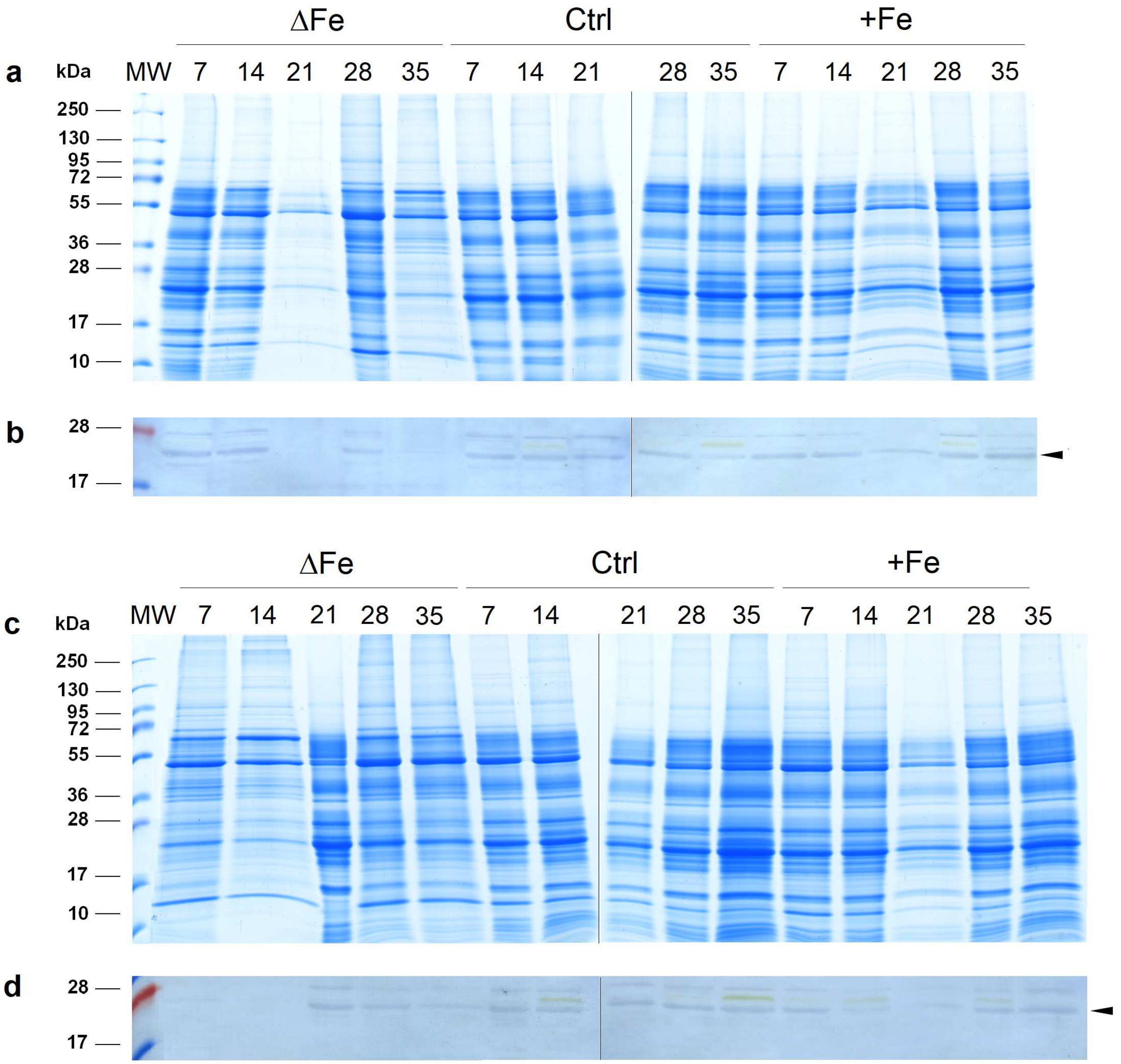

Supplementary Fig. S4 Coomassie stained SDS PAGE pattern of total chloroplast proteins isolated from 4th and 6th leaves (**a**&**c**, respectively) and immunoblot against NiCo on the identical samples (**b**&**d**, respectively). Lanes on protein gels and immunoblots were loaded with 20 µg protein except lanes belonging to the following samples: 4th leaves (A): ΔFe 21 days (4 µg); ΔFe 35 days (18 µg); +Fe 21 days (18 µg); 6th leaves (C): ΔFe 14 days (12 µg); Ctrl 21 days (11 µg); +Fe 21 days (15 µg), due to low protein concentration in the samples. The 26 kDa band (arrowheads) is identified as BnNiCo (see Supplementary Fig. S2). ΔFe – Fe deficiency, Ctrl – optimal Fe nutrition (control), +Fe – superoptimal Fe nutrition. Numbers indicate the time of treatment (days) of the isolates. As for

molecular weight standard (MW) PageRuler™ Plus Prestained Protein Ladder (Thermo-Fisher Scientific; Lot.: 00463463) was used

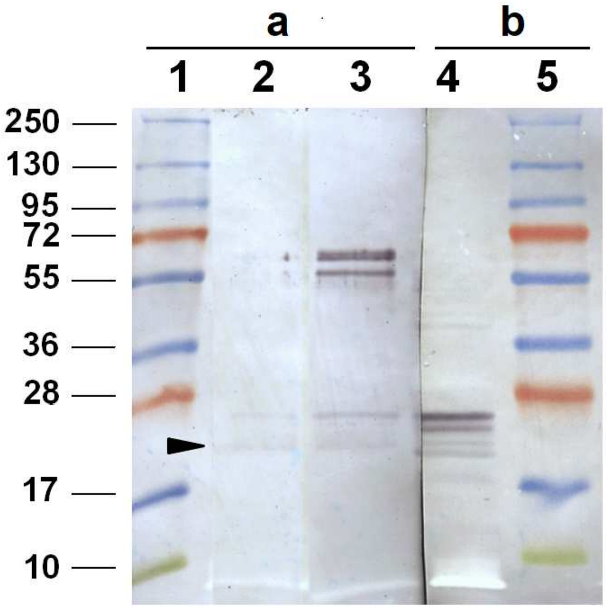

Supplementary Fig. S5. Identification of BnPIC1 protein. Marks: 1, 5 – molecular weight standard; 2 – total chloroplast proteins solubilized according to Laemmli UK (1970) Nature 227: 680; 3 – chloroplast inner envelope membrane fraction, isolated according to Solti et al. (2014, New Phytol 202: 920) and solubilized according to Duy D et al. (2007, Plant Cell 19: 986). using 250 µg ml^{-1} Pefabloc instead of PMSF; 4 – total chloroplast proteins solubilized according to Laemmli (1970). Samples were run on the same gel and blotted to the same nitrocellulose membrane. After blotting, the membrane was cut (**a** and **b**) and treated with the antibodies separately. **a** – immunoblot against PIC1 using rabbit polyclonal antibodies directed against PIC1 from *Arabidopsis*; **b** – immunoblot against LHCII using rabbit polyclonal antibodies against Light Harvesting Complex II apoprotein. As for molecular weight standards, PageRuler™ Plus Prestained Protein Ladder (Thermo-Fisher Scientific; Lot.: 00463463) was used. Lanes were loaded with 30 µg solubilised protein. Arrowhead is pointing on BnPIC1 at 21 kDa

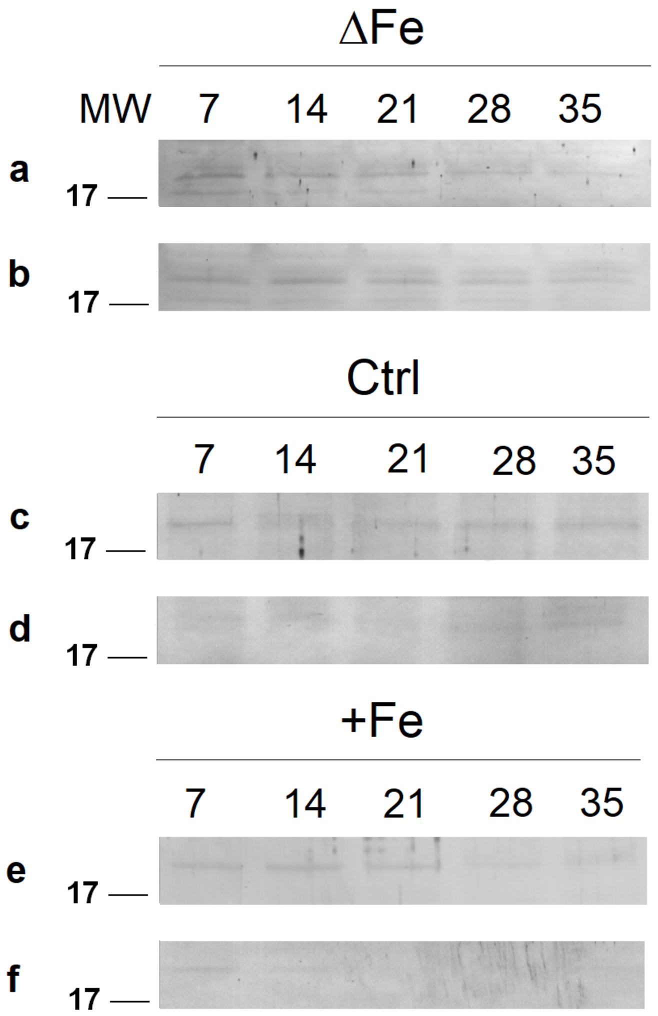

Supplementary Fig. S6 Immunoblot against PIC1. Lanes were loaded with 20 µg protein except lanes belonging to the following samples: 4th leaves (**a**): ΔFe 21 days (4 µg); ΔFe 35 days (18 µg); (**e**) +Fe 21 days (18 µg); 6th leaves (**b**): ΔFe 14 days (12 µg); (**d**) Ctrl 21 days (11 µg); (**f**) +Fe 21 days (15 µg), due to low protein concentration in the samples. The 21 kDa band (arrowheads) is identified as BnPIC1 (see Supplementary Fig. S5). ΔFe – Fe deficiency, Ctrl – optimal Fe nutrition (control), +Fe – superoptimal Fe nutrition. Numbers indicate the time of treatment (days) of the isolates. As for molecular weight standard (MW) PageRuler™ Plus Prestained Protein Ladder (Thermo-Fisher Scientific; Lot.: 00463463) was used

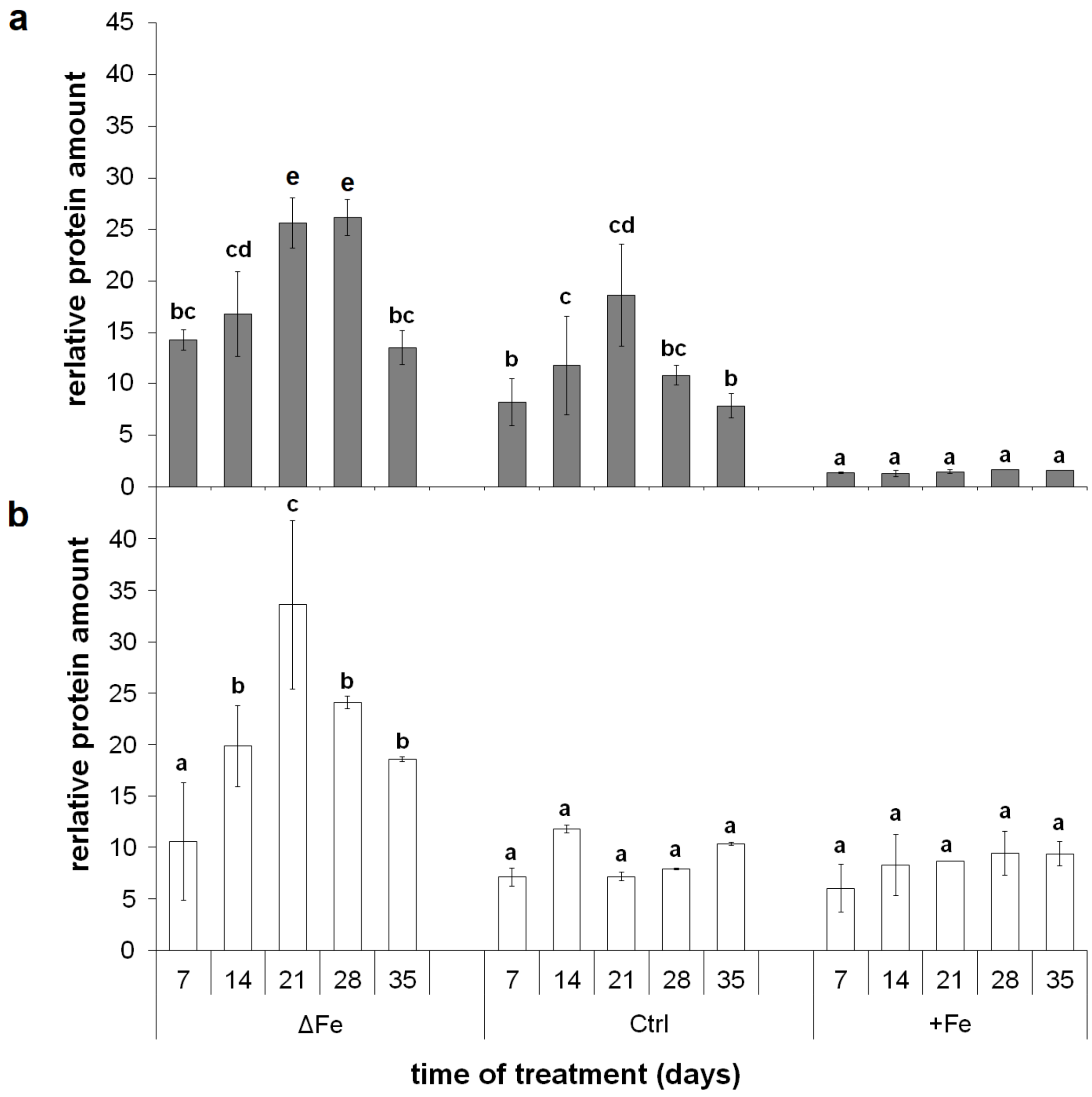

Supplementary Fig. S7 Relative amount of PIC1 protein based on immunoblot analysis; **a** – 4th leaves; **b** – 6th leaves. ΔFe – Fe deficiency, Ctrl – optimal Fe nutrition (control), +Fe – superoptimal Fe nutrition. Error bars represent SD values. To compare the differences, one-way ANOVAs were performed with Tukey-Kramer *post-hoc* tests on the treatments (P<0.05; n=3×2 [biological×technical])

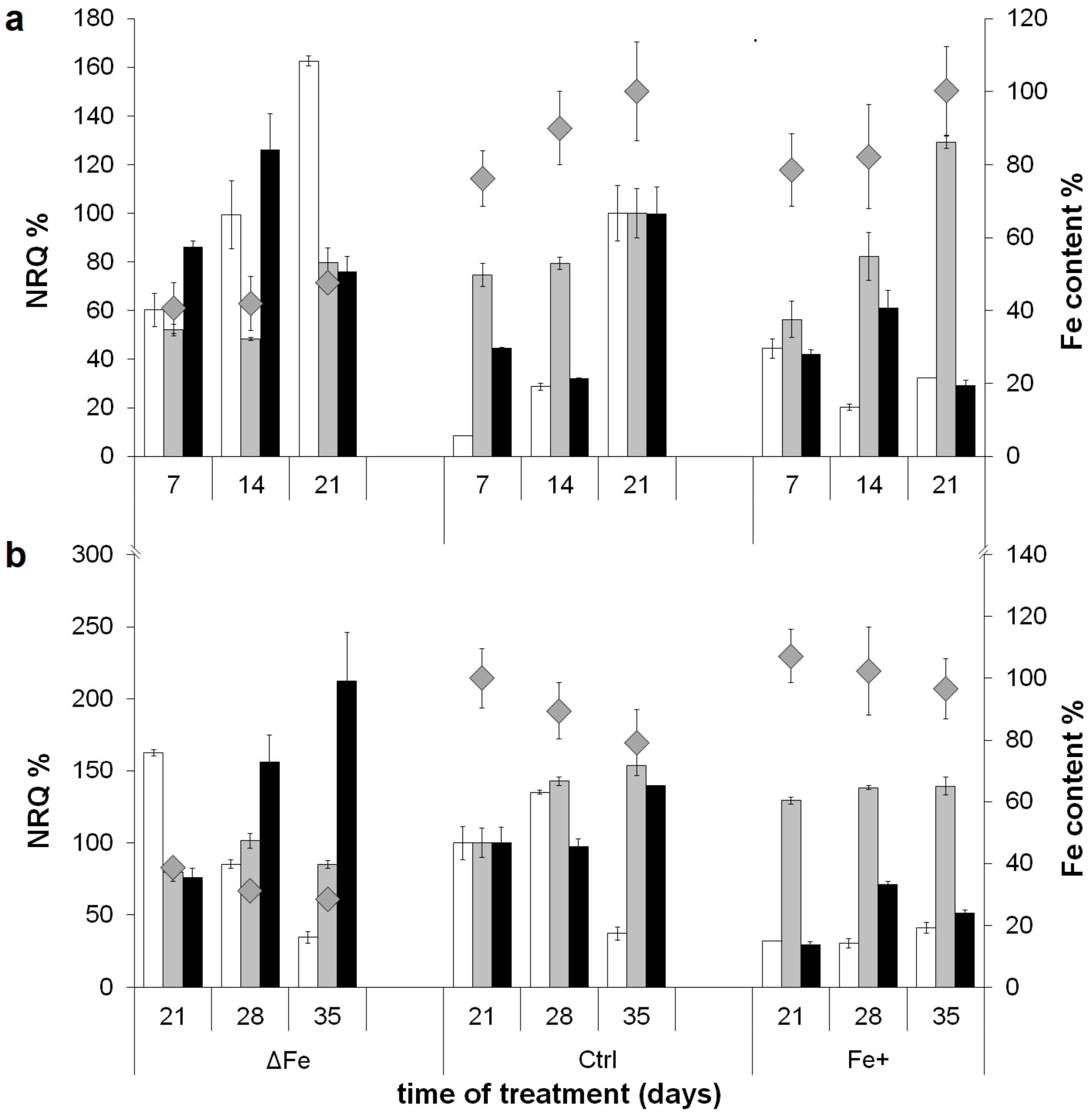

Supplementary Fig. S8 Correlation between chloroplast Fe content and the expression of GOIs during **(a)** and following (**b**) the development of leaves, based on data of 4th leaves; Ctrl, optimal Fe nutrition (control); +Fe, supraoptimal Fe nutrition. For the easier comparison of datasets, 21-day values of optimal iron nutrition plants were chosen as basis of normalisation (100%). Diamond, chloroplast Fe content; open, grey and closed columns, expression of *BnPIC1*, *BnNiCo* and *BnMAR1*, respectively
